# A randomized control trial of re-designed and shorter research informed consent forms to improve comprehension

**DOI:** 10.1177/17407745261429936

**Published:** 2026-03-28

**Authors:** Ezekiel J Emanuel, Samantha Lopez-Rico, Merjan Lijerón Ozisik, Eli Silvert

**Affiliations:** Department of Medical Ethics and Health Policy, Perelman School of Medicine, University of Pennsylvania, Philadelphia, PA, USA

**Keywords:** Informed consent, research ethics, informed consent form, equity

## Abstract

**Background/Aims:**

Informed consent forms are an essential component of ethical clinical research. However, they are failing. They are long, legalistic, and written at an 11th-grade reading level. Consequently, many participants have poor comprehension of the research they are considering. Our objective is to assess participant comprehension and satisfaction across three informed consent forms of different lengths and formats including one based on the Food and Drug Administration’s (FDA) 2024 draft summary template.

**Methods:**

In total, 334 healthy participants from AmeriSpeak’s online survey panel were recruited via email and AmeriSpeak’s member portal, oversampling less educated individuals and those from racial and ethnic minority groups. Participants were randomized to three informed consent forms—long, medium, and short—and were asked to imagine hypothetically being a patient considering joining a study. They were given a brief introduction on the purpose of informed consent forms and of this project’s goal to evaluate comprehension, satisfaction, and willingness to join the described hypothetical study but were not told the hypothesis or that there were three different informed consent forms. The long informed consent form (21 pages) was used in an actual international Bristol Myers Squibb study concerning a stroke prevention regimen. The long informed consent form contained longer paragraphs and lists and was written just below an 11th-grade level. The long informed consent form was modified to create the medium (14 pages) and short informed consent forms (3 pages), which both used simpler words, shorter sentences, less passive voice, tables, and other patient-friendly edits to achieve below sixth-grade reading levels. The short informed consent form was adapted from the Food and Drug Administration’s 2024 draft summary template.

**Results:**

The medium informed consent form (mean score of 79.3%) scored significantly higher on comprehension than the long informed consent form (65.7% mean score, p < 0.001) or the short informed consent form (69.3% mean score, p = 0.006). Compared to the long informed consent form, fewer medium informed consent form participants found their informed consent form “too long” or “too detailed.” Compared to the long and medium informed consent forms, more short informed consent form participants reported wanting more information on study procedures.

**Conclusion:**

The medium-length informed consent form, written at a sixth-grade reading level, with shorter words and sentences, tables, and enhanced formatting, produced significantly better comprehension than longer or shorter informed consent forms. The short informed consent form produced the same level of comprehension as a long informed consent form used in approved clinical trials. Researchers, pharmaceutical companies, and institutional review boards should adopt these informed consent form modifications to increase participant comprehension and satisfaction.

## Background

The informed consent process is a foundational element of ethical clinical research.^1,2^ Informed consent forms (ICFs) are critical to this entire process for three reasons. Prospective participants are routinely given and read ICFs before considering enrolling in a study. While research staff conversations are intended to expand on the written ICF and ensure that prospective participants are adequately informed about a study,^3^ the written ICFs typically guide study staff explanations and conversations with prospective participants. Written ICFs serve as the first resource for participants to consult with questions about the study when they are away from the study site. Furthermore, although conversations with research staff play an important part in informing participants, institutional review boards (IRBs) can have no prior or concomitant review, oversight, assessment, or accountability of these discussions.^4–6^ Written ICFs are the only part of the informed consent process IRBs assess and make suggestions for revision. Appropriately, ICFs have been subject to careful study and evaluation.

In practice, ICFs frequently fall short of Good Clinical Practice Guidelines for informed consent documents to be clear, concise, and avoid unnecessary volume and complexity.^7,8^ They are often excessively long, legalistic, and written at reading levels too high for the general population.^7,9^ For example, ICFs for phase III COVID-19 vaccine trials averaged over 8000 words.^10^ A 2024 study found that the average readability level of ICFs corresponds to the 11th grade,^11^ whereas the literacy skills of 54% of US adults fall below a sixth-grade level.^12^ Globally, across Organisation for Economic Co-operation and Development member countries, an average of 26% of adults demonstrate similar limited literacy.^13^ Consequently, many participants lack adequate comprehension of critical research concepts such as randomization and risk.^14,15^

Research studies since at least 1969 suggest that shortened and simplified ICFs either improve or maintain participant comprehension while increasing satisfaction.^16–21^ Despite promising indications, broad adoption of shorter and simplified ICFs remains limited. Why?

Many barriers prevent broad adoption of shorter and simplified ICFs including excessive preoccupation with legal protections by sponsors, study sites, and IRBs, inclusion of unevaluated and often unnecessary institutional boilerplate language, and lack of easily adoptable, alternative ICF templates. In addition, part of the hesitation in adopting improvements may reflect limitations of previous studies that fail to offer a clear example of an ICF template that is both rigorously tested for comprehension and can be easily implemented. First, IRBs and sponsors often limit the modifications to ICF forms that are tested.^22–24^ For instance, many IRBs insist that their boilerplate be included in any revised ICF. This boilerplate is often written at a high reading level and is untested for clarity and understanding.^24–27^ Most worrisome, it means the comparisons in multi-center studies are not of the exact same ICF, but of ICFs that differ at least by the boilerplate and often more from IRB edits.^24^ Second, many ICF improvement studies tested in clinical trials have unrepresentative participant cohorts, with few minorities and people with low educational attainment, groups that are increasingly important for Food and Drug Administration (FDA) approval.^28–31^ Third, staff interactions with patients before assessments of understanding evaluate the oral explanations, which undermines testing of the written ICF.^24,25,32^ Fourth, some studies lack a control group due to IRB preference. and others do not randomize participants to ensure consistent procedures across study staff.^30,33^ Finally, some studies pool data from multiple ongoing trials, combining results from different ICFs.^32,33^ Combining data from different ICFs in different trials makes it impossible to compare results because there is no control for variations in the ICFs.^32,33^ These limitations appear to be part of the reason adoption of shorter, more readable ICFs are limited.

Similarly, ICF improvements based on videos, multimedia, and other digital tools have not been adopted because they lack strong evidence of improving understanding, are high cost, and, unlike paper forms, may present logistic challenges in routine research processes.^32,34–37^

To overcome these limitations, the FDA recently released a draft two-page summary template to be appended to ICFs.^38^ While well intentioned, this template was written at an eighth-grade reading level. Moreover, when a short summary is added to a standard ICF, participants routinely ignore the full-length ICF before providing consent.^39^ This finding raises the question of whether the FDA’s summary template might serve more effectively as a standalone, short-form ICF.

To address the widespread concern about the complexity of ICFs,^7^ the present study aimed to overcome these limitations by evaluating, in a randomized trial, comprehension and satisfaction using three ICFs, each describing the same stroke prevention trial and could be easily implemented by sponsors and IRBs. The three ICFs included the original full-length form used in actual research, a rarely tested medium-length version, and a highly condensed version adapted from the FDA’s template. The hypothesis was that the medium-length ICF would produce the highest comprehension and satisfaction, striking the right balance between information and length.

## Methods

### Development of three ICFs

The long ICF was originally written and used by Bristol Myers Squibb in a research study spanning 30 countries from January 2019 to March 2022. The study tested a stroke prevention drug and included 2366 participants.

To create the medium ICF, the authors modified and shortened the Bristol Myers Squibb long ICF by removing repetition, simplifying language, eliminating passive voice, and shortening sentences. In addition, risk information was presented in tables, and graphic timelines of clinic visits were added. After this initial revision, CommunicateHealth, Inc., a professional health literacy firm, made additional edits to improve readability—for example, replacing “reimbursed” with “getting paid back” and “appropriate” with “acceptable”^40^ (Supplement ICFs).

The short ICF was based on the FDA’s 2024 proposed two-page summary. It achieved a lower reading level by using simpler language, minimizing passive voice, and including tables and timelines.

The University of Pennsylvania’s IRB reviewed both the medium and short ICFs to ensure regulatory compliance.

Table 1 summarizes characteristics of each ICF using Readable ContentPro. Compared to the long ICF, the medium ICF reduced the words by 44% and the short ICF by 81%. The long ICF was just below an 11th-grade reading level, while both the medium and short ICFs had reading levels under the sixth grade. The Flesch Reading Ease Score is a ranking of 0–100, with higher scores being easier to read. Scores less than 60 are difficult.^42^ The long ICF had a score under 60, while both the medium and short ICFs exceeded 60.

**Table 1.** Characteristics of three informed consent forms (ICFs).

Metric	Long ICF	Medium ICF	Short ICF
Pages	21	14	3
Reading Time^a^	31 min 28 s	17 min 30 s	6 min 6 s
Word Count	7080	3941	1376
Flesch-Kincaid Reading Grade Level (Lower is better)	10.69	5.97	5.31
Flesch-Kincaid Reading Ease^b^ (Higher is better. Over 60 being a key threshold according to HHS)	48.01	70.44	72.18

All comparisons were assessed by Readable.

a Reading Time was calculated by dividing total word count by the average reading speed of an adult, which is 238 words per minute by Readable.^41^

b Range of Reading Ease is 0 to 100, with 100 indicating the easiest to read, and scores less than 60 are considered difficult by HHS.^42^

### Participants

Participants (n = 334) were recruited from NORC’s AmeriSpeak Panel, a probability-based sample representative of US adults, drawn from area probability and US Postal Service address–based samples covering 97% of US households.^43^ The sample size was informed by an a priori power analysis with a conservative effect size using G*Power software. For the study to have a power level of 0.80, a significance level of 0.05 and an effect size of Cohen’s f = 0.19 (which corresponds to η^2^ = 0.035, meaning the ICF group explains 3.5% of the variance in scores which is roughly a half-question difference on the 14-point scale), a sample of 270 participants would be necessary.

The sample oversampled individuals with less than a high school diploma and individuals from racial and ethnic minority groups. Participants were randomly assigned to one of the three ICF groups. While a stroke study ICF was used and participants were representative of US adults, including younger adults, 10% of all stroke patients are under 40 years of age, so the hypothetical trial is still relevant to younger people.^44^

Non-patient participants were used because IRBs often restrict testing of shorter ICFs with actual patients. Prior studies have shown that IRBs commonly require inclusion of boilerplate language, averaging 463 words at an 11th-grade level.^25,26,27^ In the largest multi-site ICF improvement study, sponsors and IRBs made simplification difficult, resulting in different ICFs across sites on top of translation differences.^24^ Sampling non-patient participants allows exclusion of untested boilerplate language and enables a more controlled comparison of ICFs with varying lengths and readability.

Some studies address these challenges by administering questionnaires after improved ICFs and then providing the standard ICF before consent. While practical for IRBs, this design does not prioritize the participants’ experience.

### Administration of ICF and questionnaire

The ICF and survey were administered online. Participants read a brief description of the study informing them it was on ICFs with an explanation of what ICFs are. Participants were also told this project’s goal to evaluate their comprehension, satisfaction, and willingness to join the described hypothetical study. However, they were not told about the three specific ICF treatments being used or the study’s hypothesis about improved comprehension with the medium ICF. Participants were advised to use a computer and complete the survey during an uninterrupted time window. To encourage full reading of the ICF, timers were added to each page. As another control, participants could not navigate backward. All participants completed the same 28-item survey assessing comprehension and satisfaction. Fourteen comprehension questions were adapted from previous ICF surveys and focused on commonly misunderstood topics, such as risks and randomization (see Supplement Table 4).^15,45^ The survey was not “open book”—participants could not refer back to the ICF. Scoring was 1 point for each question, so each participant had a score out of 14. The other 14 questions were unscored and treated as categorical variables in subsequent analysis.

### Data analysis

This study was not pre-registered because it was initiated before *Clinical Trials* finalized its policy requiring pre-registration of all trials. The primary hypothesis was that the medium-length ICF would create the highest level of comprehension on the 14 comprehension questions in the survey.

Differences in mean comprehension scores across the three ICF groups were assessed through one-way analysis of variance (ANOVA). When a significant difference in means was found (i.e. p < 0.05), we compared comprehension scores in each ICF group by running three post hoc Tukey’s Honestly Significant Difference tests. In addition, differences in individual item-level responses (i.e. to questions that yielded categorical data rather than continuous data) were assessed with χ^2^ tests of independence. On the item level, when observed counts differed significantly from expected counts (i.e. p < 0.05), we compared response counts between each ICF group with three pairwise χ^2^ tests of independence. These item-level analyses were conducted for descriptive and exploratory purposes to identify content areas where differences across ICFs were most pronounced. P-values have not been adjusted for multiple hypotheses testing.

To explore whether differences in comprehension across different ICFs persisted after accounting for sociodemographic characteristics, a multivariate analysis was conducted. A logistic regression with L2 regularization was trained to predict “high” versus “low” comprehension score, with the median number of correct answers, 11, set as the threshold for “high” versus “low” scores. A regularized logistic regression was chosen because its coefficients can be converted and easily interpreted as odds ratios, it models nonlinear relationships, and it is unlikely to overfit the training data. A binary prediction task with the median score as the cutoff was chosen because our goal was to understand and easily interpret predictors of adequate versus inadequate comprehension. Sociodemographic characteristics selected to be covariates in the model, in addition to the type of ICF, were those from Table 2 that had differences in mean comprehension scores across their classifications (differences that were considered significant according to one-way ANOVA prior to adjustment for multiple hypothesis testing). These selected covariates were race (White, Black, Asian, Hispanic/Latino, or Other), education level (less than high school and high school or equivalent, some college, associates, or bachelor’s degree, or post-graduate or professional degree), and health insurance (private, government, or other), and they were converted to numerical format by creating binary indicator columns for each covariate value (a process called “one-hot encoding”).^46^ The model was trained on data from 80% of participants with fivefold cross-validation and tested on a held-out set of 20% of participants. Odds ratios were calculated based on learned covariate coefficients, and 90% confidence intervals were obtained through bootstrapping (i.e. repeating the training process 1000 times to get a probability distribution of covariate coefficients).

**Table 2.** Participants’ sociodemographic characteristics.

Characteristic	Classification	Overall N (% of 334)	Long ICF (% of 103)	Medium ICF (% of 107)	Short ICF (% of 124)
Age	Mean (lowest–highest)	51.4 (18–90)	49.3 (18–89)	52.5 (18–86)	52.1 (19–90)
Sex	Male	167 (50.0)	50 (48.5)	55 (51.4)	62 (50.0)
	Female	167 (50.0)	53 (51.5)	52 (48.6)	62 (50.0)
Race/ethnicity	White, non-Hispanic	194 (58.1)	57 (55.3)	65 (60.7)	72 (58.1)
	Black, non-Hispanic	46 (13.8)	12 (11.7)	11 (10.3)	23 (18.5)
	Asian, non-Hispanic	29 (8.7)	10 (9.7)	8 (7.5)	11 (8.9)
	Hispanic/Latino	59 (17.7)	21 (20.4)	20 (18.7)	18 (14.5)
	Other, non-Hispanic	6 (1.8)	3 (2.9)	3 (2.8)	0 (0.0)
Education	Less than high school and high school or equivalent	125 (37.4)	44 (42.7)	36 (33.6)	45 (36.3)
	Some college, associates, or bachelor’s degrees	152 (45.6)	42 (40.8)	52 (48.6)	58 (46.7)
	Post-graduate degree or professional degree	57 (17.1)	17 (16.5)	19 (17.8)	21 (16.9)
Household income	Less than $25,000	34 (10.2)	13 (12.6)	7 (6.5)	14 (11.3)
	$25,000-$49,999	69 (20.7)	27 (26.2)	23 (21.5)	19 (15.3)
	$50,000-$99,999	129 (38.6)	35 (34.0)	41 (38.3)	53 (42.7)
	$100,000 or more	93 (27.9)	25 (21.3)	33 (40.9)	35 (28.2)
Geography	Urban	124 (37.1)	47 (45.6)	35 (32.7)	42 (33.9)
	Suburban	159 (47.6)	38 (36.9)	58 (54.2)	63 (50.8)
	Rural	51 (15.3)	18 (17.5)	14 (13.1)	19 (15.3)
Region	Northeast	60 (18.0)	22 (21.4)	12 (11.2)	26 (21.0)
	South	111 (33.2)	35 (34.0)	38 (35.5)	38 (30.6)
	Midwest	71 (21.3)	20 (19.4)	22 (20.6)	29 (23.4)
	West	92 (27.5)	26 (25.2)	35 (32.7)	31 (25.0)
Prior experience in research studies	0	281 (84.1)	85 (82.5)	91 (85.0)	105 (84.7)
	1 or more	44 (13.2)	12 (11.7)	14 (13.1)	18 (14.5)
	Undisclosed	9 (2.7)	6 (5.8)	2 (1.9)	1 (0.8)
Form of health insurance	Private	168 (50.3)	50 (48.5)	54 (50.5)	64 (51.6)
	Government program	135 (40.4)	39 (37.8)	45 (42.0)	51 (41.1)
	Other	31 (9.3)	14 (13.6)	8 (7.5)	9 (7.2)

All analysis was done with Python (version 3.11.7). SciPy (version 1.11.4) was used for statistical tests, and scikit-learn (version 1.2.2) was used for modeling.

## Results

### Sociodemographic characteristics of the study sample

The mean age of participants was 51.4 years (range = 18–90) (Table 2). Fully 37.4% had a high school education or less, 40.2% were minorities, and 84.1% had no prior experience in a research study. These demographic characteristics were consistent across the three ICF groups (Table 2).

### ICF effect on overall comprehension

Of 14 comprehension questions, the medium ICF group scored 79.3% (11.1 answers) correct, significantly higher than 65.7% (9.2) for the long ICF (p < 0.001) and 69.3% (9.7) for the short (p = 0.006) (Table 3). There was no significant difference in mean scores between the long and short ICFs (p = 0.62).

**Table 3.** Overall comprehension and satisfaction scores by ICFs.

Topic	Overall	Long ICF	Medium ICF	Short ICF	p-value
Mean Comprehension Score (% correct out of 14 questions)	10.0 (71.4)	9.2 (65.7)	11.1 (79.3)	9.7 (69.3)	Overall: < 0.001 Long versus Medium: < 0.001 Long versus Short: 0.620 Medium versus Short: 0.006
Number who felt Well Informed or Very Well Informed (%)	318 (95.2)	96 (93.2)	103 (96.2)	119 (95.9)	Overall: 0.520
Felt they received all the information they wanted, n (%)	270 (80.8)	88 (85.4)	85 (79.4)	97 (78.2)	Overall: 0.352
Read the ICF Very Carefully or Carefully, n (%)	255 (76.3)	78 (75.7)	85 (79.4)	92 (74.1)	Overall: 0.627
ICF was Too Long, n (%)	74 (22.2)	41 (39.8)	13 (12.1)	20 (16.1)	Overall: < 0.001 Long versus Medium: < 0.001 Long versus Short: < 0.001 Medium versus Short: 0.580
ICF was Too Detailed, n (%)	43 (12.9)	27 (26.2)	4 (3.7)	12 (9.7)	Overall: < 0.001 Long versus Medium: < 0.001 Long versus Short: 0.002 Medium versus Short: 0.130
ICF was Hard or Moderately Hard to understand, n (%)	40 (12.0)	16 (15.5)	12 (11.2)	12 (9.7)	Overall: 0.854
ICF was Easy or Moderately Easy to understand, n (%)	219 (65.6)	55 (53.4)	74 (69.2)	60 (72.6)	Overall: 0.515
Very Unlikely or Unlikely they would participate in the study, n (%)	154 (46.1)	58 (42.3)	52 (48.6)	59 (47.6)	Overall: 0.238
Very Likely or Likely they would participate in the study, n (%)	73 (21.9)	30 (29.1)	20 (18.7)	23 (18.6)	Overall: 0.273

The medium ICF had a greater number of correct responses than the long ICF for five questions (Q4, Q6, Q10, Q11, Q13) and more correct responses than the short ICF for three questions (Q9, Q10, Q12) (Supplement Table 4). There were no questions where the long or short ICFs significantly outperformed the medium ICF.

Regarding commonly misunderstood topics (e.g. risk), the medium ICF was consistently equal or better. When asked to name the most common risk of the drug, participants with the medium ICF answered the question correctly 68.2% of the time—substantially more than the 31.1% correct by the long ICF (p < 0.001) but similar to the 57.3% by the short ICF (p = 0.11) (Supplement Table 4). For randomization, 73.8% of the medium ICFs were correct which was better than 57.3% of the long ICF (p = 0.017) but similar to 71.0% of the short ICF (p = 0.074).

Based on the multivariate analysis, participants who were more likely to have high comprehension were those who read the medium ICF (odds ratio (OR) = 1.57; 95% confidence interval (CI) = 1.28–1.95), post-graduate or professional level of education (OR = 1.31; 95% CI = 1.20–1.73) and were White, non-Hispanic (OR = 1.43; 95% CI = 1.23–1.83) (Supplement Table 5).

### ICF effect on satisfaction

Among the medium ICF group, 12.1% reported the ICF was “too long.” This was substantially less than the 39.8% of participants reporting the long ICF was too long but similar to the 16.1% who found the short ICF too long (Table 3). Similarly, 3.7% of the medium ICF group felt the ICF was “too detailed,” fewer than the 26.2% for the long ICF while similar to the 9.7% for the short ICF. There were no noteworthy differences across the ICF groups for the other satisfaction metrics (Table 3).

### Participant recommendations for ICF improvement

Overall, across the three ICFs, there were no major differences in participants wanting a specific improvement (Supplement Table 6). Regarding study procedures, 2.9% for the long ICF, 9.3% for the medium ICF, and 14.5% for the short ICF wanted more information. In total, 13.6% of participants who read the long ICF recommended having less information on information privacy compared to 4.7% for the medium ICF and 1.6% for the short ICF.

## Discussion

A medium-length ICF, written at the sixth-grade level, with concise and patient-friendly language, shorter sentences, less passive voice, tables, timeline graphics, and enhanced formatting produced significantly better comprehension of a clinical research study than longer or shorter ICFs. Six points need emphasis.

First, these results suggest that the medium ICF strikes the balance between being concise—it was 56% the word count of the long ICF—and having sufficient detail for greater comprehension of key concepts. Simultaneously, fewer participants found it “too long” or detailed. Conversely, participants did not feel that the medium ICF was “too long” or “too detailed” compared to the short ICF. Regarding the length and depth of specific sections, the long ICF found a substantially higher proportion of participants feeling that there was excessive detail on privacy and data protection. In addition, a higher proportion of participants who read the short ICF wanted more information regarding study procedures compared to the medium and long ICFs. Thus, the medium ICF displays an optimal amount of information without being deemed too long or detailed.

Second, the long and detailed ICF did not lead to better comprehension of fundamental concepts. Compared to the long ICF, the medium and short ICF cohorts had a better grasp of widely misunderstood concepts such as randomization and risk. The medium and short ICF may find increased comprehension in these areas because they incorporated tables, graphics, and a shorter description of randomization with clearer analogies. Importantly, for the medium ICF, the proportion of correct answers regarding randomization and risks (73.8% and 68.2%, respectively) exceeds that reported in previous global studies assessing comprehension (53.1% and 38.5%, respectively).^15^

Third, the FDA draft guidance-based short form was not optimal as a standalone ICF. A three-page ICF adapted from the FDA’s draft summary produced the same level of comprehension as a long ICF used in an approved clinical trial.^22–24,28–33^ However, the short ICF failed to maximize participant comprehension and satisfaction that the medium ICF achieved.

Fourth, this study indicates that implementing patient-friendly techniques should be adopted as standard in all ICFs. Despite data suggesting shorter ICFs produce better comprehension, uptake has not occurred. This study provides a head-to-head comparison of three ICFs with statistically significant data showing that a medium ICF using good writing techniques, tables, and timeline graphics can improve comprehension. Unlike other interventions, the medium ICF can readily be adopted without changing current research and IRB workflows or increasing costs.

These data should also overcome resistance by IRBs, lawyers, and others. IRBs might be persuaded to decrease or eliminate their boilerplate and extra details since participants found the extensive privacy boilerplate of the long ICF too detailed, were less satisfied with the long ICF, and since the long ICF did not maximize comprehension. Furthermore, because the long ICF did not maximize comprehension, it should be seen as a less useful approach to reduce liability threats. Another approach to shorten and clarify ICFs to improve comprehension would be for the FDA and other regulators to mandate use of tables for risks and benefits, timelines, clear analogies for randomization, and other simple graphics. In light of these results, pharmaceutical companies might change their ICF templates. Indeed, that both Bristol Myers Squibb and Johnson & Johnson have already implemented elements of this medium ICF into their global templates suggests that having data and easily implemented ICF improvements can finally lead to change.^47^

Fifth, shortening the ICF might improve the discussion between study staff and prospective participants. While this study did not assess these conversations, written ICFs typically provide the “script” used by staff to describe the study to prospective participants. Having shorter ICFs with illustrative graphics may make these discussions more focused and could support participant comprehension of critical information that is frequently misunderstood such as randomization and risks.

Finally, there is great need and pressure to increase the racial and educational diversity of participants in trials. Studies enroll populations that tend to be more educated than average. This may very well be because long ICFs with details which prospective participants find excessive may dissuade minorities or less educated individuals from reading them and enrolling. In this study, the greatest predictor for increased comprehension was whether participants were assigned the medium ICF rather than the long or short ICF. While the medium ICF did not lead to greater comprehension among lower-educated and minority participants, it did not worsen their comprehension. Furthermore, very few participants in this study desired “more information,” suggesting the detail is counterproductive. This finding calls into question the added value of longer ICFs with substantial detail.

### Limitations

This study has six limitations. First, the sample consisted of healthy U.S. adults rather than actual research participants. However, no ICF improvement study in a real trial has achieved both a large, representative cohort and the ability to test a single ICF. In large multi-site trials, IRBs require site-specific boilerplate language and are often translated into other languages, resulting in different ICFs that preclude true apples-to-apples comparisons.^24^ Single-institution studies allow for consistent ICFs but have small sample sizes.^22,23,28–33^ Moreover, patient samples often reflect the demographics of the study sites or disease area, not the general population or broader trial-eligible groups.^28–31^ Using healthy participants enables testing the same ICF with a large, demographically representative sample.

Second, this study did not assess participants’ comprehension of the entire informed consent process—reading the ICF and discussing with study staff. It is possible discussions eliminate all differences in comprehension based on different ICFs. However, as noted, IRBs can only review ICFs, not such discussions with study staff. ICFs also typically structure the information discussed by study staff, and ICFs are what patients can repeatedly and most quickly consult when away from the study site. Furthermore, it may well be that many prospective participants do not enroll even before talking with study staff because of poor ICFs. Thus, the written ICFs have significant impact on the entire informed consent process and should be improved even if they are not the only part of the process.

Third, healthy volunteers may behave differently than patients with illnesses who may benefit from enrolling in a research study. Healthy participants may either not read the information closely enough or read it closer than patients. However, these participants were told comprehension would be assessed, and three quarters reported reading the ICF carefully. Informed consent research exhibits a constant tension between testing an ICF in simulated or real clinical settings.

Fourth, there is no standardized comprehension questionnaire for ICFs. Although there have been attempts to create a standardized metric for comprehension, the complexities and differences of each trial make a generic questionnaire unfeasible.^45^ However, there are concepts such as randomization, risks, and placebo that are consistently tested within the literature. The questionnaire included these concepts as well as trial requirements, such as tests and length of the study. In addition, questionnaires may readily assess recall rather than comprehension. The two are difficult to separate but are correlated.^48^ Although questionnaires may be flawed in capturing true comprehension, they are widely used in the literature and allow for some comparison across studies over time.

Fifth, this study only examined a single type of clinical trial. There are other types of studies that may entail greater complexity such as cancer research. ICFs for more complex studies may be more difficult to shorten or simplify. However, the medium ICF demonstrates that it is not necessarily the length that maximizes comprehension but the inclusion of concise and patient-friendly language as well as tables and timeline graphics. Information in complex studies can still be written in a way that facilitates understanding and minimizes jargon.

Finally, the ICFs were administered online which may not replicate reading a paper ICF. For example, paper ICFs allow participants to go back and forth and gauge the true length of the form. However, after they were randomized, participants were told the approximate length of time of the session. In addition, as health care becomes more digital, online ICFs, or those read on a screen, are likely to become the norm.

## Conclusion

ICFs for research studies are excessively long and written at high reading levels, resulting in low participant comprehension. Yet inertia, aversion to shorter forms, costs, and lack of alignment among stakeholders preclude adoption of ICFs which improve comprehension. This study found that a medium ICF, which was 56% the length of a normal ICF and written at a sixth-grade reading level with simpler words, active voice, shorter sentences, risk tables, and improved formatting, leads to greater comprehension overall and of commonly misunderstood concepts. Researchers, pharmaceutical companies, and IRBs should adopt these ICF modifications to increase participant comprehension and satisfaction.

## Supplemental Material

sj-docx-1-ctj-10.1177_17407745261429936 – Supplemental material for A randomized control trial of re-designed and shorter research informed consent forms to improve comprehension Supplemental material, sj-docx-1-ctj-10.1177_17407745261429936 for A randomized control trial of re-designed and shorter research informed consent forms to improve comprehension by Ezekiel J Emanuel, Samantha Lopez-Rico, Merjan Lijerón Ozisik and Eli Silvert in Clinical Trials

sj-docx-2-ctj-10.1177_17407745261429936 – Supplemental material for A randomized control trial of re-designed and shorter research informed consent forms to improve comprehension Supplemental material, sj-docx-2-ctj-10.1177_17407745261429936 for A randomized control trial of re-designed and shorter research informed consent forms to improve comprehension by Ezekiel J Emanuel, Samantha Lopez-Rico, Merjan Lijerón Ozisik and Eli Silvert in Clinical Trials
