# Supplementary material for: A randomized control trial of re-designed and shorter research informed consent forms to improve comprehension
Source: Clin Trials. 2026 Mar 28;23(4):421–9. doi: 10.1177/17407745261429936 (PMC13451594; doi:10.1177/17407745261429936)
Supplement: Supplementary file 1 [file sj-docx-1-ctj-10.1177_17407745261429936.docx]

**Survey Questions**

**Informed Consent Experiment**

Conducted for: BMS

Conducted by: NORC at the University of Chicago

Sample Source: AmeriSpeak Probability-Based Panel

Sampled Population: General Population 18+

Date Fielded: September 20^th^, 2024-October 21^st^, 2024

## WINTRO_1.

This survey is about informed consent for medical research.

[SHOW IF P_COND = 1]

## DISPLAY_GOAL_C.

**Project Goal**

Informed consent forms are important documents used in medical research studies. These forms tell the patient considering whether to join a study about general tests and procedures, risks and side effects, and their right to leave the study.

Using a modified informed consent form, this study will measure your comprehension, satisfaction, and willingness to join a possible drug study. We hope these findings will inform researchers on how to improve the informed consent form and the entire process.

[SHOW IF P_COND = 1]

## DISPLAY_INSTRUCTIONS_C.

**Instructions**

We are asking you to help improve the informed consent form that patients will read before participating in a drug testing study. Before deciding to join the study, a patient would read this informed consent form.

Imagine you are one of these patients. You will read the informed consent form and answer some questions. Be aware that the form might be long; it could take you about 40 minutes to read. Many of the pages are time-restricted to ensure that you have read everything.

After reading the form, you will answer about 30 short questions. We expect this part to take a maximum of 5 to 10 minutes.

We suggest that you take this survey **only on a laptop or computer** and **when you have 45 minutes,** to avoid interruptions or distractions. If that isn’t now, feel free to close the survey and re-enter it later.

**Thank you for reading this question thoroughly, you will be able to move on to the next page after one minute.**

[SHOW IF P_COND = 1]

## DISPLAY_INFOPART_C

Bristol Myers Squibb, CV010031, Revised Protocol 06, 09Oct2020

**Informed Consent Form**

### INFORMATION TO PARTICIPANTS

| NAME OF STUDY: | A Global, Phase 2, Randomized, Double-Blind, Placebo-Controlled, Dose-Ranging Study of Astraxo, an Oral Factor XIa Inhibitor, for the Prevention of New Ischemic Stroke or New Covert Brain Infarction in Patients Receiving Aspirin and Clopidogrel Following Acute Ischemic Stroke or Transient Ischemic Attack (TIA) |
| --- | --- |
| STUDY NUMBER: | CV010031 |
| STUDY SPONSOR: | Bristol Myers Squibb Company  Route 206 & Province Line Road, Lawrenceville, NJ 08543 USA  Avenue de Finland 4, B-1420 Braine-l’Alleud, Belgium  6-5-1 Nishi-Shinjuku, Shinjuku-ku, Tokyo, 163-1327,  Japan  (Co-developed under a collaboration agreement between Bristol Myers Squibb and Janssen Pharmaceuticals, Inc.)  EU Legal Sponsor address:  Bristol Myers Squibb International Corporation Chaussée de la Hulpe 185, 1170 Brussels, Belgium |
| STUDY DOCTOR (INVESTIGATOR): | Dr. John Smith  111-111-1111 |

[SHOW IF P_COND = 1]

## DISPLAY_INFOEX_C.

**Why Are You Receiving this Information?**

You are invited to participate in this clinical research study because you have recently had a stroke or mini- stroke. The following information describes the study and your role as a possible participant. Please read this information carefully, and do not hesitate to ask your study doctor any questions to ensure that you are able to make an informed decision as to whether to participate.

**What Is the Purpose of this Clinical Research Study?**

Stroke and mini-strokes, called transient ischemic attacks (TIAs), are conditions where blood clots form in the vessels of the brain and can block blood circulation. This can result in serious disabilities or death. Blood thinners work by stopping clotting proteins from binding together or platelets from adhering to one another to prevent blood clots from forming. The purpose of this study is to determine if an investigational study medication, Astraxo, which inhibits factor XIa (one of the components in the clotting process) is safe and effective in preventing future strokes when given daily with the antiplatelet medication clopidogrel and aspirin or aspirin only for up to 90 days.

Previous testing of Astraxo in humans has been promising and treatment was found safe and well tolerated. It has been tested in 1529 humans to date. It is expected that approximately, and up to, 2350 subjects will be enrolled in this study to evaluate the effectiveness of Astraxo with antiplatelet medication versus antiplatelet medication alone.

**Thank you for reading this question thoroughly, you will be able to move on to the next page after one minute.**

[SHOW IF P_COND = 1]

DISPLAY_STUDYINVOLVE_C.

**What does the study involve?**

You will be randomly assigned to either group: 50 mg Astraxo twice daily or placebo (inactive treatment that looks like Astraxo).

Astraxo will be given to you as capsules in a blister pack. You will take 4 capsules each day from the blister pack (which is one full row of the blister pack). You are to take 3 capsules in the morning and 1 in the evening. You will also take 1 tablet of aspirin 100 mg and 1 tablet of clopidogrel 75 mg every day during your morning dose for the first 21 days, and for days 22 to 90 you will take 1 tablet of aspirin. Neither you nor your study doctor will know if you receive Astraxo or placebo, but the treatment with aspirin and clopidogrel is open and known.

Study participation includes 1 study visit while in the hospital, 2 follow-up study visits at the study site, and 2 study-related telephone calls over the next 3 months. Participation also includes 2 study MRIs and additional blood samples. Further details on the required study visits can be found in**Part A** of this consent form.

It is expected that approximately, and up to, 2350 subjects will be enrolled in this study.

During the study, we will also collect and record information about you. Your personal details such as date of birth, ethnicity, and gender will be collected as well as medical history, prior and current medication(s) and the results of study specific procedures and tests.

If you participate in the study, you must follow all the instructions given regarding the intake of other medicines. This applies to common drugs that are given on prescription, medicines that can be bought without a prescription and natural remedies. Your doctor will discuss this with you.

If you do not wish to participate in the study or cancel your participation, you will receive the treatment that your study doctor believes will be the best for you. Your doctor will, if necessary, explain to you different treatment options and inform you of the possible benefits and disadvantages of these.

**Thank you for reading this question thoroughly, you will be able to move on to the next page after one minute.**

[SHOW IF P_COND = 1]

## DISPLAY_ADVANTAGES_C

### Potential advantages, disadvantages and possible risks:

You will be given close attention from the study staff during the time you are involved in the study. If the investigational study medication combination is effective, you may benefit by a reduced risk of having a recurrent stroke. It is possible that you may not personally benefit from your participation in this study, but by taking part, you will provide new information that may benefit other patients in the future.

All drugs may cause certain side effects and discomforts.

[SHOW IF P_COND = 1]

## DISPLAY_ASTRAXO_C

### Astraxo

Astraxo is a blood thinner (anticoagulant) which as a class of drug is associated with a risk of bleeding. It may occur as minor bleeding, such as nosebleed, bleeding gums, or bruising. More serious bleeding may occur in critical organs of the body such as in the brain, stomach, or bowel (intestine). Increased bleeding may also happen during surgery or invasive procedures. A serious bleeding event can be dangerous, permanently disabling, or fatal.

Based on what we have learned up to this point about Astraxo, different types of bleeding events have been reported:

- Mild bleeding: nosebleed, bleeding gums, small amounts of blood in the stool, bruising and bleeding at an injection site (area where a shot or needle had been given)
- Serious bleeding leading to hospitalization: bleeding from the stomach, bleeding in the brain within the area of a previous stroke and bleeding during surgical operation

These bleeding events happened while patients were either taking Astraxo alone or with other blood thinner medications. Therefore, the role of Astraxo in these events cannot be excluded or confirmed.

Other most frequent side effects reported with Astraxo include headache, loose stools, nausea, dizziness, rash, abdominal discomfort, and back pain.

All drugs have a potential risk of an allergic reaction, which could become life threatening. You should stop Astraxo/placebo and seek medical help immediately if you think that you have any of the following symptoms of a serious allergic reaction: trouble breathing; swelling of the face, mouth, lips, gums, tongue, or neck; or seizures. Loss of consciousness, shock and death may result from heart and lung failure in rare cases.

Of note, there was a case of a rash leading to hospitalization in a patient taking multiple medications in addition to Astraxo. Although the drug may not have caused the rash, its contribution cannot be excluded at this time.

Stop taking Astraxo/ placebo and call your study doctor right away if the rash involves any of the following conditions:

- Rash that covers a large area of your body
- Rash is painful or there are blisters
- You have sores (lesions) in other areas such as eyes, inside the mouth or nose, vagina, urethral opening, glans penis, and the anus
- Symptoms experienced shortly before the rash such as sudden appearance of malaise (not feeling well), unusual tiredness, fever (measure your temperature), loss of appetite, stinging, redness, or burning of eyes, sore mouth or difficulty swallowing

There may be risks with the use of Astraxo that are not yet known. During the study, the sponsor will learn new information about the study drugs and the risks. It is possible that this new information might make you change your mind about being in the study. If new relevant information is discovered, your study doctor will tell you about it in a timely manner.

**Thank you for reading this question thoroughly, you will be able to move on to the next page after two minutes.**

[SHOW IF P_COND = 1]

## DISPLAY_ADDITIONALRISK1_C

### Additional Risks

There are possible risks of interactions (like increased bleeding) between Astraxo and other medications, such as the ones listed below. You must review all current medications at the beginning of the study and inform the study doctor if there are any planned new medications. If you are taking any of the medications listed below or are asked to start them during the study, you must discuss this with your study doctor immediately. You should not take these medications during this study unless the medication is given by your doctor as one of the study medications.

- Salicylates (Aspirin® and Aspirin®-containing products)
- NSAID (non-steroidal anti-inflammatory drug) use longer than 14 days such as Motrin®, Aleve®, Advil®
- Omeprazole or esomeprazole for the duration of the clopidogrel treatment
- Other blood thinners such as:
  - Vitamin K antagonists (eg, warfarin, Coumadin®)
  - Direct oral anticoagulants (eg, Xarelto® [rivaroxaban], Eliquis® [apixaban], Pradaxa® [dabigatran])
  - Clopidogrel (eg, Plavix®)
  - Ticagrelor (eg, Brilinta®, Brilique®)
  - Prasugrel (eg, Effient®)
- Medications that can affect CYP3A4 (an enzyme in the body that breaks down Astraxo)
  - antifungals (for example itraconazole, ketoconazole, or posaconazole)
  - antibiotics (for examples clarithromycin, telithromycin or rifampin)
  - HIV medication (for example, ritonavir, indinavir, nelfinavir, atazanavir, saquinavir, or cobicistat)
  - anti-convulsant (for example phenytoin or carbamazepine)
  - antidepressant (nefazodone)
  - herbal medicine: St. John’s wort

Do not consume grapefruit juice during the study as potentially it can interact with the study medication.

**Thank you for reading this question thoroughly, you will be able to move on to the next page after one minute.**

[SHOW IF P_COND = 1]

## DISPLAY_ADDITIONALRISK2_C

For the combination of **low dose aspirin** **(Aspirin^®^)** **and clopidogrel(Plavix^®^),**the following side effects may occur:

*Common (1-10%):*

- Easy bruising/bleeding, bleeding that may take longer to stop, nosebleeds, blood in your urine (pink, red, or brown-colored urine), coughing up blood or blood clots, bleeding from the stomach or rectum (signs include feeling faint, vomiting blood or vomit that looks like coffee grounds, having bloody or black stools) or having stomach pain that does not get better)

*Uncommon (<1%) side effects include:*

- Bleeding from the stomach or rectum, heartburn, nausea, vomiting, sensitivity reaction (e.g., skin rash, hives, itching, difficulty breathing/asthma, or swelling of your face, lips, tongue, or throat)

In rare cases the combination of low-dose aspirin and clopidogrel can increase your risk of serious and potentially life-threatening bleeding (e.g., bleeding in the brain).

Clopidogrel treatment can *rarely (0.01-0.1%)* cause a blood-clotting problem called thrombotic thrombocytopenic purpura (TTP). In TTP, blood clots form in blood vessels anywhere in the body and can potentially be life-threatening and symptoms include the following: Purplish spots on your skin/mouth due to bleeding under the skin, yellowing of your skin or the whites of your eyes, tiredness or weakness, pale-looking skin, fever, fast heart rate or shortness of breath, seizure, nausea, vomiting or diarrhea, vision loss.

Alcohol can increase your risk of bleeding while you are taking clopidogrel.

When taking **Aspirin** the following side effects may occur:

*Common (1-10%):*

- upset stomach, stomach pain,
- heartburn
- easy bruising/bleeding.

*Uncommon (<1%):*

- nausea, vomiting
- ringing in your ears
- allergic reaction (skin rash, hives, itching, or breathing difficulties)
- bleeding from the stomach (signs include feeling faint, vomiting blood, having bloody or black stools/bowel movement, or having stomach pain that does not get better).

You must be able to tolerate low-dose aspirin and clopidogrel to participate in this study. Aspirin can be found in many over-the-counter and prescription medicines. In addition, aspirin, clopidogrel and Astraxo can interact with numerous prescription and over-the-counter medicines and herbal and natural products (e.g. St.John’s wort). You must tell the research team about all medications you are taking during the study including over-the-counter medications. The research team may ask you to stop taking certain medicines, continue using medications, or to use other medications. Discuss with your study doctor before taking any pain-relieving medications.

**Thank you for reading this question thoroughly, you will be able to move on to the next page after one minute.**

[SHOW IF P_COND = 1]

## DISPLAY_ADDITIONALRISK3_C

**Other Possible Risks and Discomforts Related to Study Procedures**

When study medications are taken alone or in combination with other medications, there may be other risks that are unknown. There is the potential that any unknown risk could be serious or fatal.

- **Blood Collection:** You may experience some temporary discomfort, pain, swelling, bruising, or (in rare circumstances) infection at the puncture site. The blood pressure cuff may also cause discomfort or bruising to the upper arm. The blood samples required to be taken during the study are detailed in **Part A** of this consent form.
- **MRI (Magnetic Resonance Imaging) Testing:** The examination is painless and takes about 30 minutes. There are no known harmful effects from exposure to the magnetic field or radio waves used in MRI imaging. Some MRI scanners are very narrow. **Talk to your study doctor if you have metal in your body** (such as heart pacemaker or prostheses), if you are uncomfortable in small spaces, or have had difficulties with a MRI or CT scan in the past. During the MRI examination, it can be very loud. You will receive headphones to reduce the noise.
- **EKG Tests:** Sticky patches will be applied to your skin, which may cause minor discomfort when attached or removed; skin irritation is rare but could occur during an EKG from electrodes or gel that is used.

**Thank you for reading this question thoroughly, you will be able to move on to the next page after one minute.**

[SHOW IF P_COND = 1]

## DISPLAY_VOLUNPART_C

### Voluntary participation and opportunity to withdraw their consent

Participation in the study is voluntary. If you wish to participate, sign the consent statement on the last page. You can withdraw from the study at any time and without giving any reason, without it having any consequences for your further treatment.

If you agree to participate in the study, you have the right to have access to information about you. If you withdraw from the study, no more information or material will be collected from you. Information already collected will not be deleted. If you later wish to withdraw or have questions for the study, you can contact your study doctor, Dr. John Smith (111-111-1111).

[SHOW IF P_COND = 1]

## DISPLAY_PERINFOSAMPLES_C

### What will happen to the samples and your personal information?

Your blood samples and the information recorded about you will only be used as described in the intent of the study. Your samples will not be used for whole genome sequencing. The samples collected during this study will be used for the purposes described in this consent form section throughout their usable life and will be destroyed at the end of the scheduled storage period. This, which will be no longer than 25 years after the end of the study or the maximum allowed by applicable law.

All information and samples will be processed without a name or other directly identifiable information. A code links you to your information and your blood samples through a name list. The list that can link your name to the code will only be kept at the study site/ hospital and only personnel responsible for the study will have access to it.

Personal information that may link you to the study (signed patient information and identification list) will be retained for at least 25 years after the final report and then deleted. It will not be possible to identify yourself in the results of the study when published.

[SHOW IF P_COND = 1]

## DISPLAY_QCONTACT_C

### Who Can You Contact with Further Questions?

You may ask questions about this consent form or the study at any time. If you have additional questions, or experience a research-related injury, contact the study doctor using the details provided in the table on the first page of this information sheet.

If you have a complaint or question about your rights as a research subject, you may contact the Ethics Committee (EC) or Institutional Review Board (IRB) using the details provided in the table on the first page of this information sheet. This is a group of scientific and non-scientific individuals who review research studies keeping the safety and welfare of research subjects in mind.

Your own research results will not be given back to you. A description of this clinical study will be available on websites, including <https://clinicaltrials.gov/> and [https://eudract.ema.europa.eu/.](https://eudract.ema.europa.eu/) These websites do not include any information that can identify you. At most, the website will include a summary of the study results. You can search this website at any time.

This website only shows data in English, but you can request information from the study staff at any time and have access to data that are publicly available.

Further information about the study can be found in **Part A.**

Further information about biobank, data privacy, finance and insurance can be found in **Part B.**

The declaration of consent follows **Part B.**

**Thank you for reading this question thoroughly, you will be able to move on to the next page after one minute.**

[SHOW IF P_COND = 1]

## DISPLAY_ALTPROCE_C

**PART A – FURTHER EXPLANATION OF WHAT THE STUDY INVOLVES**

### Alternative procedures or treatment

If you decide not to participate in this study, you will receive the standard treatment(s) for stroke or TIA. Standard treatments include blood clotting inhibitors, at the discretion of your doctor. The important potential risks for the standard treatment are bleeding and recurrent stroke, and benefits for the standard treatment are known.

### Study Schedule – what happens and when does it happen?

**Screening Phase:**

Before you can participate in the study, you will need to sign this informed consent form. Then the study staff needs to find out if the study suits you.

- Treatment with Astraxo will not begin until after you have met all the study criteria and completed the study MRI.
- You will need to have a CT or MRI scan of your brain and vascular imaging of the arteries in your brain and neck.
- A complete physical examination will be performed (including height and weight, and vital signs, such as heart rate, and blood pressure) and you will have an EKG (electrocardiogram of your heart).
- The study doctor will perform a number of assessments to evaluate signs and symptoms of your condition.
- Your medical history, current diagnosis including routine laboratory results and current medication will be reviewed.
- You may have blood samples taken for additional laboratory tests if needed.
- A urine or serum pregnancy test will be required if you are a woman and capable of becoming pregnant.
- You will need to stay at the hospital overnight to be there for a total of 2 days.

All tests and assessments are done to make sure that it is safe for you to participate in the study. There is a possibility that the results of these tests show that you’re not a good candidate for this study. In this case you will **not** continue with any study activities after screening.

However, if you agree to participate and your study doctor determines that you are a good candidate for the study, additional procedures will be performed according to the following schedule:

**Thank you for reading this question thoroughly, you will be able to move on to the next page after one minute.**

[SHOW IF P_COND = 1]

## DISPLAY_TREATMENTPHASE_C

### Treatment phase:

At the **Day 1 Visit in the hospital,** you will have a study-specific MRI of your brain unless already taken. The study doctor will perform a number of assessments to evaluate signs and symptoms of your condition.

- You will be provided with the Astraxo study medication, clopidogrel and aspirin, and instructions on how and when to take your medication.
- You must follow all instructions given regarding the intake of study medications: clopidogrel (morning): no special requirements, aspirin (morning): intake after meal with enough water, not to be taken with alcohol, Astraxo (morning and evening): no special requirements. Tablets of clopidogrel, aspirin and Astraxo should not be split or crushed.
- You will have blood drawn for safety laboratory testing to assess your health status.
- You will have a series of blood draws to monitor drug levels of Astraxo in your body (PK) while you are in the hospital, along with other samples to check the effect of the study medication on the body (PD) and exploratory biomarkers related to the associated biological pathways, neurological diseases, cardiovascular diseases, SARS-CoV-2 infection status (virus that causes COVID-19) and related conditions.

[SHOW IF P_COND = 1]

## DISPLAY_DAY2160_C

At the **Day 21 Visit at the study site,** a number of assessments to evaluate signs and symptoms of your condition will be performed, including:

- Physical examination, vital signs, EKG, blood tests
- You will be asked about the medication(s) you are using and any symptoms or events that might have occurred in the meantime
- Additional blood samples will be collected (for safety, PK, PD and biomarkers)
- You will be asked to return all used and un-used study medication (blinded study medication, study clopidogrel and study aspirin) that were dispensed to you during the previous visit; the site staff will review them and redispense the unused aspirin and Astraxo/placebo medication. You will also receive new study medication supplies. You will be asked about your compliance with the study medication taken since you left the hospital.

On **Day 30** if you are a woman who can get pregnant you will be asked to perform a pregnancy test at home and report the date and results (with a kit that you should have received from your previous visit at the study site).

On **Day 60**the following information will need to be obtained via a telephone call:

- You will be asked about any medication changes since your last visit, changes in your health or new symptoms, and compliance with the study medication since the last visit.
- If you are a woman and capable of becoming pregnant, you will be asked to perform a home pregnancy test and report the date and results (with a kit that you should have received from your previous Day 21 visit at the study site).

**Thank you for reading this question thoroughly, you will be able to move on to the next page after one minute.**

[SHOW IF P_COND = 1]

DISPLAY_DAY90_C

At the **Day 90/ End Of Treatment Visit at the study site,** the study doctor will perform a number of assessments to evaluate signs and symptoms of your condition, including:

- MRI must be done either on the scheduled Day 90 visit. You must still take study medication until the MRI is performed (maximum days on study medication: 96).
- Physical examination, body weight and vital signs, and EKG will be performed
- Compliance with study medication and changes to daily medications will be evaluated and your study doctor will ask questions about side effects or symptoms experienced since the telephone visit
- Blood samples will be taken (for safety, PK, PD and biomarkers) and a pregnancy test will be performed if applicable
- You will be asked to return all study medication that was not taken UNLESS you have not completed the end of study MRI.

In case you have stopped the study medication early, the assessments above will be conducted at an End-of- Treatment visit except the originally scheduled Day 90 MRI. Afterwards, site staff might contact you for a follow-up telephone contact between the last date of study medication dose and the originally scheduled Day 90 MRI.

[SHOW IF P_COND = 1]

DISPLAY_DAY97_C

**Day 97 Visit** (one week after the Day 90 MRI):

You will be **contacted by telephone** by one of the study staff members to discuss how you feel and any symptoms or events that you have potentially experienced since you performed the Day 90 MRI.

During the study, you might be asked to return to the site for additional blood draw(s) if important laboratory values are out of normal range or if a sample could not be measured by the laboratory. These re-tests are done for the purpose of your own safety.

In the event that you are unable, or not allowed to attend a visit to the study site due to unavoidable circumstances (e.g. COVID-19 pandemic), other measures can be considered to obtain the required visit assessments as local regulations allow. Examples include but are not limited to, telephone calls, videoconferencing, use of local imaging or laboratory facility for assessments, home visits, shipment of study drug/assessment kits directly to your home and/or use of other facilities for study visits. The study staff members will discuss the specific allowed measures with you and your acceptance to follow them.

[SHOW IF P_COND = 1]

DISPLAY_ENDSTUDY_C

### What Will Happen at the End of the Study?

At the end of study, or if you have decided to no longer take the study medication before its conclusion, you will be required to complete an End-of-Treatment Visit to return study medication and complete a safety check followed by an End of Study Phone call one week after you have performed the Day 90 MRI. After the study medication is stopped, your study doctor or primary care physician will decide what medical treatment you should continue to receive.

There may be a need to obtain information about how you are feeling after the study is completed, to further assess the safety and efficacy of the study treatment. This can mean that you are contacted again by study staff by telephone or letter. If your study doctor or study nurse cannot get in touch with you, he / she can contact the person you have stated as relatives, your General Practitioner, your local hospital where you can obtain information through public registers that doctors have access to. The study staff may also use publicly available sources such as registries, newspaper and social networking websites or may ask for the assistance of a third-party representative and may share with that representative limited information about you, such as your name and last known address. The representative will consult public sources, such as public health registries and databases, to obtain your current contact information. The representative will only share this information with the study doctor, to help him or her complete the follow-up stage of the study. This does not happen if you withdraw your consent to participation.

**Description of Pharmacokinetic Testing (PK):**

During your hospital stay, 3 blood samples are expected to be taken relative to your first or second dose of study medication. At Day 21 and Day 90 visits, an additional blood sample will be collected. In addition to these times, a blood sample will be collected within 48 hours of a new stroke event or serious bleed. These blood samples will be processed for the purpose of examining how much drug is in your blood stream and what your body does to the drug. In addition, residual plasma samples may be used for other assessments.

**Description of Pharmacodynamic Testing (PD):**

During your hospital stay, a blood sample will be drawn prior to first dose of study medication and 3 blood

samples are expected to be collected relative to your first or second dose of study medication. At the visits on Days 21 and 90, an additional blood sample will be collected. In addition, to these times, a blood sample will be collected within 48 hours of a new stroke event or serious bleed. The goal of these assessments is to explore the effect of the study medication on your body, your illness, and similar medical conditions.

### Thank you for reading this question thoroughly, you will be able to move on to the next page after two minutes.

[SHOW IF P_COND = 1]

## DISPLAY_BLOODVOLUME_C

### What is the required blood volume that will be taken during the study?

A total of approximately 200 mL (40 teaspoons) of blood will be collected during the whole study. The amount of blood taken each time will be about 10 mL (approximately 2 teaspoons).

This blood volume will be used for:

- Routine safety laboratory testing to check on your health
- PK blood markers to assess how Astraxo moves through your body (approximate total volume: 20 mL - about 4 teaspoons)
- PD blood markers to measure the effect of Astraxo on your body and exploratory biomarkers that can aid in understanding if and how your body will respond to treatment (approximate total volume: 120 mL – about 24 teaspoons)
- At any relevant bleeding or a new stroke event about 21 mL of blood will be collected (approximately 4 teaspoons).

[SHOW IF P_COND = 1]

## DISPLAY_RESPONSIBLITIES_C

### What are your responsibilities as a patient and study participant?

- To commit the time required to keep appointments on Days 1, 21 and 90, receive telephone calls on Days 60 and 97, and complete the Day 1 and Day 90 study MRI assessment.
- To tell the study doctor about your complete medical history including current medications.
- To report any concerns, illnesses, or changes in medication you take during the study.
- To take and return study medication as instructed. You are expected to return unused study medication and empty containers at every visit to the study site.
- To tell your study doctor or study staff if you change your address, telephone number, or other contact information, even if you determine that you no longer want to actively participate in the study.
- **To carry your Subject Alert Card with you at all times during study participation.**
- To sign a separate medical release form to obtain safety information in the case that you are hospitalized during the study.

[SHOW IF P_COND = 1]

## DISPLAY_REPRORISK_C

### What Are the Reproductive Risks?

In pregnant rats and rabbits given Astraxo, there was no effect on the fetus (unborn baby). Some side effects (e.g., inflammation around the eyes, reduced food consumption) were observed in pregnant rabbits that were given very high doses of Astraxo. The relationship of this finding to humans taking Astraxo is uncertain. It is not known if the study treatment may have an effect on male or female fertility, an unborn child or nursing infant. For that reason, females capable of becoming pregnant and male participants with female partners that are capable of getting pregnant, **MUST** use an acceptable method of birth control throughout the entire study. Please discuss questions about acceptable methods of birth control with your study doctor. The type of birth control you use must be discussed with your study doctor before you begin the study. Your study doctor must approve the method you use before you can enter the study.

[SHOW IF P_COND = 1]

## DISPLAY_FEMALEPART_C

### Female participants in the study:

If you are breastfeeding, pregnant, or plan to become pregnant, you may **NOT** participate in this study. It is important to use **approved contraception during the study period until 30 days after the last dose of study medication.**

Pregnancy tests will be done in women - capable of getting pregnant - before the start of study and during the study.

However, should a pregnancy occur during this time, you should immediately stop taking the study medication and contact your study doctor. You / your partner and your child will be taken care of in the usual way within the local health service. With your permission, your study doctor may ask you to collect and access your medical records and the records for your infant after the delivery. Pregnancies occurring up to 30 days (according to study Sponsor requirement) after the last dose of the study drug must also be reported to the study doctor. The study Sponsor will not pay for routine medical care relating to your pregnancy.

[SHOW IF P_COND = 1]

## DISPLAY_MALEPART_C

### Male participants in the study:

For male participants with female partner of childbearing age, it is important that you inform your spouse / partner that you are participating in this study. In addition, you cannot donate semen during this period. It is important to use **approved contraception during the study period until 30 days after the last dose of study medication**.

It is important that you contact your study doctor if your partner becomes pregnant while you are in the study until 30 days after your last dose. She may be requested to sign a separate informed consent form for the collection of data about the pregnancy and the outcome of the pregnancy if required by country regulation.

You can be asked to inform your study doctor about the outcome of the pregnancy. Your partner and the baby will be taken care of in the usual way by the local health service.

[SHOW IF P_COND = 1]

## DISPLAY_THREEQUARTER-C

**Almost there! You are now over three quarters of the way through the informed consent form.**

[SHOW IF P_COND = 1]

## DISPLAY_ACCEPTEDBC_C

### Acceptable Methods of Birth Control:

**For Female Participants:** If you are sexually active and able to become pregnant, you must use an approved safe method for birth control during the study. The study doctor will discuss this with you.

Examples of birth control methods acceptable in this study include:

- Combined (estrogen- and progestogen-containing) hormonal contraception associated with inhibition of ovulation
  - oral
  - intravaginal
  - transdermal
  - injectable
- Progestogen-only hormone contraception associated with inhibition of ovulation
  - oral
  - injectable
- Spiral
- Diaphragm with spermicide
- Cervical cap with spermicide
- Vaginal Sponge with spermicide
- Abstinence for the entire duration of the study, if this is your preferred and usual lifestyle
- Menopause - last 12 months
- Surgical sterilization of you or your male partner

Women using a hormonal method of birth control (such as birth control pills, patch, or injectables) should notify the study doctor if they are taking any prescription drugs, including over-the-counter drugs or herbal supplements, as they may reduce the effect of hormonal birth control.

### For Male Participants:

- Condoms
- Abstinence
- Surgical sterilization

[SHOW IF P_COND = 1]

## DISPLAY_NEWINFO_C

### New Information Becomes Available during the Study

Your study doctor will inform you or your legal representative in a timely manner of any new information learned during the study that may affect your willingness to continue your participation in the study.

[SHOW IF P_COND = 1]

## DISPLAY_CHANGEMIND_C

### What Happens if You Change Your Mind?

Your participation in this study is voluntary. You do not have to take part, and you may discontinue your involvement at any time without penalty or loss of benefits to which you are otherwise entitled. If you decide to leave the study before the last study visit, tell the study doctor and follow his or her instructions. It may be helpful if you could explain your reasons. You may receive standard treatment and no prejudice will be shown towards you for medical care or participation in future research. Please note that any information collected before your withdrawal will be kept and used to complete the research.

If you decide to stop taking the study medication, your study doctor will not presume that you have withdrawn from the study but will assume that you will continue to participate in any follow-up activities, as described in the section for study procedures. If you decide to stop taking the study medication for any duration of time or no longer wish to come to the study site for scheduled visits, please inform your study doctor as soon as possible. Stopping study medications without medical supervision could put you at risk. It is very important that you discuss alternate treatment options with the study doctor before you stop taking study medications suddenly.

If your participation in the study is stopped early, you may be asked to complete the end of study procedures (such as a final medical examination and laboratory tests) for your own safety. If you do not want to participate in any of the follow-up activities, you must inform your study doctor in writing if possible, and clearly identify the activities you do not want.

Please note that even if you withdraw consent for further follow-up or contacts, if your study doctor becomes aware of additional safety information, this will be reported to the study Sponsor in order to comply with legal and regulatory requirements.

**Can Someone Else End Your Participation in the Study?**

Your study doctor or the study sponsor, FDA, and IRB may end your participation in the study at any time, for several reasons, including but not limited to:

- Your study doctor feels that it is in your best interest to stop participating in the study
- You experience side effects that are difficult to manage
- The study is stopped early
- You or your partner become pregnant
- Any other reason that may affect your overall health
- You are unable to make your scheduled appointments
- Administrative reasons such as the sponsor stopping development of Astraxo

Keep in mind that even if your participation is ended early, any information that has already been collected from you like the research data will still be used to complete the study and will still be maintained by the study sponsor.

**Thank you for reading this question thoroughly, you will be able to move on to the next page after two minutes.**

[SHOW IF P_COND = 1]

## DISPLAY_PARTB_C

**PART B – PRIVACY, INSURANCE**

### Are There Any Costs if You Decide to Participate?

The study drugs will be made available to you at no charge, and you will not be required to pay for any study procedures. You or your insurance company may be billed for any standard medical care that is not required for the research study.

### Is There a Payment if You Decide to Participate?

You will not receive payment for participating in this study, but the study drugs will be made available to you at no charge and you will not be required to pay for any study procedures. You may be reimbursed for any reasonable travel expenses (bus/train/taxi fares) incurred as a result of taking part in this study on production of a receipt.

### Will You Receive Compensation if You Are Injured as a Result of the Study?

If you are injured during your participation in this study, you should contact the study doctor as soon as possible in person or at the telephone number listed on page one of this consent form. Medical care may be obtained in the same way you would ordinarily obtain other medical treatment.

If you suffer a study-related injury, the reasonable and customary costs of necessary medical treatment of the injury will be reimbursed to the extent that these costs are not covered by your private insurance or other third-party coverage. Please contact the study doctor regarding reimbursement for a study-related injury.

A study-related injury is a physical injury that is directly caused by the study drug administered as described in the study protocol.

A study-related injury does not include injuries directly caused by any of the following:

- The natural course of your underlying disease or medical condition
- Not following the instructions provided in this consent form or by study staff

There are no plans to provide any other payments or other forms of compensation for a study-related injury (for example, for lost wages or discomfort). You do not give up any legal rights by signing this consent form.

**Medicare, Medicaid, and TRICARE Beneficiaries:**

If you are a Medicare, Medicaid, or TRICARE beneficiary, the costs of necessary medical treatment of the study-related injuries described above may not be billed to Medicare, Medicaid, and TRICARE. These medical bills should be submitted first to study doctor for review and payment. Contact your study doctor if you have any questions about this restriction.

**Thank you for reading this question thoroughly, you will be able to move on to the next page after two minutes.**

[SHOW IF P_COND = 1]

## DISPLAY_DATA_C

### What Will Happen to Your Data?

This research study may be performed only by collecting and using your medical information. Your study records will be kept as confidential as possible. *Only a number and your initials will be used to identify you*. You will not be personally identified in any reports or publications that may result from this research study. Because of the research goals of this study, however, your study records cannot be kept completely confidential. The Sponsor of this study is Bristol Myers Squibb. Bristol Myers Squibb has contracted PPD, a contract research organization, to manage this study.

[SHOW IF P_COND = 1]

## DISPLAY_CONFISTOR_C

### Confidentiality, Collection, Storage, and Use of Study Data Collected during the Study

The study staff, the study Sponsor and its agents, and PPD will need to review the medical information collected from you for use in this study in order to accurately record information for this study. In addition, in order to review the study findings, the US FDA and other regulatory agencies may review your medical records. The following sections provide a specific description of how your information will be used and disclosed if you participate in this research study. By signing this consent form, you are authorizing such access, on site or through other means of remote monitoring approved by the Sponsor and as local regulatory requirements permit, to review your medical records remotely (outside the facilities of the study site, either by providing redacted copies, by granting secure access to the electronic systems or via a telehealth system). If you do not sign this form to authorize access, you will not be able to participate in this research study.

### Collection of Study Data

Your study doctor and study staff will collect information about you that is relevant to this study; specifically, information about your name, address, contact details, date of birth, images, health, race, ethnic origin, life habits, and sexual life. This collected information about you is called “data” or “study data” in this document. You will be asked to sign a separate medical release form to obtain safety information in the case that you are hospitalized during the trial.

### Confidentiality of Study Data and Key-Coded Data

For the purposes of your participation in this study and the protection of your identity, your study doctor will assign you a unique code, such as a series of numbers and/or letters. The study doctor will record the study data collected from you in a report form that uses your assigned code, not your name. This is to protect your study data by making it anonymous for most study purposes.

The data that are recorded with your assigned code rather than your name is called “key-coded data”. The key-coded data will be entered into the study’s computer database. Your study doctor will keep a confidential list linking your name to your code, and only authorized persons will have access to this list. The ways in which key-coded data may be used and shared is described below in following section. Some study data will identify you (such as medical records), and the ways in which these data may be used and shared is described below in the section **Use and Sharing of Study Data that Identify You**.

### Use and Sharing of Key-Coded Data

Your key-coded data may be shared with and used by the following:

- The study Sponsor, its current or future research partners, collaborators, assignees, licensees or designees and their affiliates, agents, and employees
- Other individuals and organizations that analyze or use your information in connection with these research activities, including laboratories and study sites (in the event you transfer to another study site)
- Domestic or foreign health authorities e.g., the FDA, and
- Institutional Review Boards

Your key-coded data will be used in connection with this study and may also be used by the parties listed above:

- for other current or future research involving the same drug(s), the same or related health conditions, or for other relevant health research
- in support of domestic or foreign regulatory filings, for example, as part of any marketing authorization application or supplement
- transferred to individuals or companies located outside of the country or region in which you reside. However, all access to the key coded data will be controlled in accordance with applicable laws and regulations. This may include written agreements that require that the data be kept confidential and secure and be used only for the purposes permitted by this consent form or applicable laws and regulations
- in publications about this study but it will remain coded. Your identity will not be revealed in any compilation, study report, or publication at any time

### Your Access to and Correction of Study Data that Identify You

You have the right to obtain any initial and updated information about the study data that identify you, as well as the right according to local law and procedures to require the correction of any errors.

This information, as well as the fact of your participation in this study, can also be provided or made known to your primary physician if you wish.

You can discuss this further with your study doctor, who will be your primary contact person for your access rights.

**Thank you for reading this question thoroughly, you will be able to move on to the next page after two minutes.**

[SHOW IF P_COND = 1]

## DISPLAY_STMTCONSENT_C

**Statement of Consent**

- I have read and understand the statements in this informed consent form.
- I have had the opportunity to ask questions and I am satisfied with the explanations provided.
- I voluntarily agree to take part in this study.
- I understand that I and/or my legal representative will receive a copy of this signed and dated written consent form.

TREATMENT SECTION

[SHOW IF P_COND=2]

## DISPLAY_GOAL_T

**Project Goal**

Informed consent forms are important documents used in clinical studies. These forms tell the patient considering whether to join a study about general tests and procedures, risks and side effects, and their right to leave the study.

Using a modified informed consent form, this study will measure your comprehension, satisfaction, and willingness to join a possible drug study. We hope these findings will inform researchers on how to improve the informed consent form and entire process.

[SHOW IF P_COND=2]

## DISPLAY_INSTRUCTIONS_T

**Instructions**

We are asking you to help improve the informed consent form that patients will read before participating in a drug testing study. Before deciding to join the study, a patient would read this informed consent form.

Imagine you are one of these patients. You will read the informed consent form and answer some questions. Be aware that the form might be long; it could take you about 20 minutes to read. Many of the pages are time-restricted to ensure that you have read everything.

After reading the form, you will answer about 30 short questions. We expect this part to take a maximum of 5 to 10 minutes.

We suggest that you take this survey **only on a laptop or computer** and **when you have 25 minutes**, to avoid interruptions or distractions. If that isn’t now, feel free to close the survey and re-enter it later.

**Thank you for reading this question thoroughly, you will be able to move on to the next page after one minute.**

[SHOW IF P_COND=2]

## DISPLAY_INFORMEDCONSENTFORM_T

**Informed Consent Form**

| **Study Title:** | Stroke Prevention Drug Astraxo with Aspirin and Clopidogrel |
| --- | --- |
| **Protocol Number:** | CV010031 |
| **Sponsor:** | Bristol Myers Squibb |
| **Study Doctor (Investigator):** | Dr. John Smith 111-111-1111 |

We understand that making a choice to join a research study can be hard. This document is an **informed consent** form. It can help you make your decision. Here are some important things to keep in mind:

- This form tells you what may happen if you decide to join this study, including the risks and benefits of joining this study.
- It is your choice to join this research study. Feel free to talk with your family and caregivers if you need help deciding.
- If you have any questions about this study, please ask the study doctor or study team (staff working with your study doctor) at the study site.
- If you want to join this study, there is a chance you may not be selected because you do not meet the requirements.
- If you join this study, you can leave at any time.
- If you choose **not** to join or if you leave this study, your study doctor will discuss your other options. You will not lose any benefits that you already have.
- You do not have to pay any money to join this study. But you may have to cover costs that come up during the study, like traveling to see your study team.

**Thank you for reading this question thoroughly, you will be able to move on to the next page after one minute.**

[SHOW IF P_COND=2]

## DISPLAY_GENELRAINFO_T

### General Information

**1.1 What is the purpose of this study?**

This study is designed to help us learn whether the study drug Astraxo works to prevent future strokes when taken daily with Aspirin and another stroke medication called Clopidogrel. Experts are still researching Astraxo to understand its side effects and how well it works. The study drug Astraxo is not approved for preventing strokes in combination with Aspirin and Clopidogrel by the Food and Drug Administration (FDA).

**1.2 Why is the study team asking me to join this study?**

You could meet the requirements to join this study because you recently had a stroke.

**1.3 Who is paying for this study?**

A company called Bristol-Myers Squibb (BMS) is paying for this study. Bristol-Myers Squibb is a company that makes medicines.

**1.4 Who is overseeing this study?**

Your study doctor oversees everyone who joins this study. Bristol-Myers Squibb will pay the study doctor for their work.

**1.5 How many people will be in this study?**

There will be about 2350 people in this study around the world.

**1.6 What are my options if I do not join this study?**

If you decide not to join this study, you can talk to your study doctor about other options. Your study doctor will direct you to the current standard treatment for preventing strokes. Aspirin and Clopidogrel are two drugs which are the current standard treatment for preventing stokes. They have risks but their benefits are known.

**Thank you for reading this question thoroughly, you will be able to move on to the next page after one minute.**

[SHOW IF P_COND=2]

## DISPLAY_TIMELINE_T

### Study Timeline, Visits, and Tests

**2.1 How long will the study take?**

From start to finish you can expect to be in this study for about 100 days.

**2.2 What are the different parts of the study?**

There are 3 separate parts of the study.

1. **Screening****:** The study team will decide if you can join the study by asking you for information and giving you different tests (screenings) like an MRI scan. You will have a 2 day stay in the hospital. If any of the screenings show that you are not the right fit to be part of the study, your study doctor will explain why.
2. **Treatment:** For people who can continue in the study, the next step is getting the study drug Astraxo with Aspirin and Clopidogrel. This part of the study will last about 90 days. Over that time, you will have about 3 in-person visits at the study site or hospital and 1 phone call with the study team.

- **Day 1 – Visit in the hospital**
- **Day 21 - Visit at the study site**
- **Day 30 – If you are a woman who can get pregnant, you will take an at-home pregnancy test**
- **Day 60 – Phone call check-in**
- **Day 90 – End of Treatment – Visit at the study site**

3. **Follow-up:** After treatment ends, you may stop getting the study drug — but you will still get a call from your study team. This part will happen 7 days after your last treatment visit (Day 90) . **It is important for your study team to know how you are feeling and how the study drug(s) are affecting you during this time.**

**2.3 What will happen during my study visits?**

At each study visit, the study team will collect your height and weight and do a physical exam.

Your study team will also do tests, including:

- MRI scans of your brain and arteries: On **Day 1**(unless already taken) and **Day 90**
- EKGs of your heart. An EKG maps your heart beat: On **Day 1, 21, and 90**

**2.4** **What kinds of samples will my study team collect?**

Your study team will collect blood samples to:

- Check how healthy you are and check for infections
- Monitor the drug levels of Astraxo
- Check the effect of Astraxo inside your body

Different amounts of blood may be taken on different visits. You will have about 200 mL of blood taken during the 100 days of the whole study.

**Thank you for reading this question thoroughly, you will be able to move on to the next page after two minutes.**

[SHOW IF P_COND=2]

## DISPLAY_TREATMENT_T

**Treatment**

**3.1 What treatments could I get during the study?**

During the treatment part, you **will** get either:

 50 mg twice a day of the study drug Astraxo **for 90 days**:
 OR a placebo, which is a dummy, sugar pill— it looks like the real pill but has no medicine in it.

During the treatment part, you **will** also take the current standard treatment:

- **For the first 21 days,** 1 tablet of 100 mg Aspirin and 1 tablet of 75 mg Clopidogrel every morning
- **For days 22 to 90**, just 1 tablet of 100 mg Aspirin

In this study you will get the study drug or the placebo based on chance — like a lottery or the flip of a coin or picking a card out of a hat. This means that both you and your study doctor will **not** know which drug(s) you’re getting, except in an emergency.

You and your study doctor will know that you are taking Aspirin and Clopidogrel.

**3.2 How will I get the study drug?**

You will take all the medicines including Astraxo, Aspirin, and Clopidogrel by mouth. Your study doctor and team will give you a pack of pills.

**Pill Schedule Table:**

| Morning | Tablet of Aspirin and Clopidogrel after a meal  Tablet of Astraxo or placebo |
| --- | --- |
| Evening | Tablet of Astraxo or placebo |

**Thank you for reading this question thoroughly, you will be able to move on to the next page after one minute.**

[SHOW IF P_COND=2]

## DISPLAY_SIDEEFFECTS_T

# **Side Effects and Other Risks**

**4.1 What are the risks of joining this study?**

Each person’s experience is different. For example, you may have side effects from:

- The study drug itself
- Taking the study drug at the same time as other drugs you need
- Study tests

If you are pregnant or breastfeeding, being in this study may also lead to health risks for your baby.

In addition, joining this study may mean that:

- You do not get the best known treatment for preventing strokes — for example, the study drug may not work or you could be assigned to take a placebo (a “dummy drug” with no medicine)
- You cannot join studies for other drugs or treatments

Talk with your study doctor if you have any questions about your options.

**4.2 What are the possible side effects of the study drug? How serious are they?**

Your study team knows about some of the side effects of the study drug, Astraxo, because 1529 people have already taken it. It is possible that there are other side effects that the study team does not know about yet. In the table below, you will find some of the possible side effects of Astraxo.

|  |  | **Side effects** |
| --- | --- | --- |
| **Very Common**  More than  1 in 10 people (10%) may have these side effects | Minor | Headache, loose stools, nausea, dizziness, stomach pain, and back pain |
|  | Serious | NONE |
| **Common**  Between 1 in 100 people and 1 in 10 people (1% to 10%) may have these side effects | Minor | Easy bruising, nosebleeds, bleeding gums, small amounts of blood in your stool, rash |
|  | Serious | NONE |
| **Rare**  Fewer than 1 in 100 people (1%) may have these side effects | Minor | NONE |
|  | Serious | Bleeding from the stomach and intestine, and bleeding in the brain which can lead to death  Serious rash that covers the entire body and is painful. Sores around the eyes, mouth, nose, vagina, urethral opening, penis, and the anus. These can be painful and stinging and cause problems swallowing. Unusual tiredness, fever, loss of appetite, redness and burning of the eyes may also happen before the rash.  Serious allergic reaction: trouble breathing, swelling of the face, mouth, lips, gums, tongue, or neck or seizures. Loss of consciousness, shock, and death from heart and lung failure |

Stop taking Astraxo or placebo and call your doctor right away if you have a rash that becomes serious.

**Thank you for reading this question thoroughly, you will be able to move on to the next page after two minutes.**

Aspirin and Clopidrogel may also have side effects. Your study doctor will discuss the side effects of each of the other drugs with you. In the table below, you will find some of the possible side effects:

|  |  | **Side effects** |
| --- | --- | --- |
| **Very Common**  More than  1 in 10 people (10%) may have these side effects | Minor | NONE |
|  | Serious | NONE |
| **Common**  Between 1 in 100 people and 1 in 10 people (1% to 10%) may have these side effects | Minor | Upset stomach, stomach pain, heartburn, easy bruising, nosebleeding, bleeding gums, small amounts of blood in stool and urine, coughing up blood or blood clots |
|  | Serious | NONE |
| **Rare**  Fewer than 1 in 100 people (1%) may have these side effects | Minor | Nausea, vomiting, ringing in your ears, allergic reaction |
|  | Serious | Bleeding from the stomach and rectum, and bleeding in the brain  Thrombotic thrombocytopenic purpura (TTP) – purplish spots on your skin and mouth, yellowing of your skin or eyes, tiredness, pale-looking skin, fever, fast heart rate or shortness of breath, seizure, vision loss |

Tell your study doctor about any side effects or changes to your health.

This will help the study team better understand if it is safe for you to continue the study, how the Astraxo works, and what other people can expect if they take Astraxo in the future.

If you have any signs of serious illness or injury, get emergency medical care right away.

**4.3 How will I learn if new information about the study drug comes out while I am part of the study?**

If your study doctor learns new information that is important to share with you, they will let you know and answer your questions about it.

**Thank you for reading this question thoroughly, you will be able to move on to the next page after one minute.**

[SHOW IF P_COND=2]

## DISPLAY_BABYRISK_T

# **Risks to a Baby**

**5.1 Could the study drug harm my baby if I get pregnant? What if I am breastfeeding?**

**Yes.** Astraxo can be dangerous to an unborn baby if you get pregnant during the study or if you are already pregnant.

The study team does not know for sure but it can also be dangerous to your baby if you are breastfeeding while getting Astraxo.

It can also be dangerous if you get someone pregnant while you are taking Astraxo.

**5.2 What do I need to do to prevent pregnancy during the study?**

You will need to use birth control that is safe every time you have sex from the time you start getting the study drug. You will find lists of appropriate types of birth control below. There are other types of birth control, but they may not work as well to prevent pregnancy. Talk with your study doctor about the type of birth control that works best for you.

You will need to take birth control during the 100 days of the study and 30 days after the last dose. Good types of birth control are:

- Birth control pills
- Birth control implant
- IUD (intrauterine device)
- Having your “tubes tied” (sterilization)
- Not having sex at all

If you have only 1 sex partner and that person has had a vasectomy (male sterilization), you may not need to use any other types of birth control during the study. Please make sure you discuss this with your study doctor.

You cannot donate eggs while you are required to use birth control.

**Appropriate types of birth control for males:**

You will need to take birth control during the entire study period 100 days and 30 days after the last dose.

- Condoms
- Having a vasectomy (male sterilization)
- Not having sex at all

Be sure to tell your study doctor if:

- Your partner gets pregnant during the study
- You have vaginal sex without a condom

You must not donate sperm during the study.

**5.3 What happens if I get pregnant during the study?**

Tell your study doctor right away if you get pregnant while you are taking the study drug — or up until 30 days after your last dose. Most of the time, your study doctor will tell you to stop taking the study drug. Then you and your study doctor will talk about your pregnancy, including options for your future care.

Your study doctor will also ask you to sign a separate consent form so the study team can collect information on the pregnancy and your baby’s health at birth — and possibly after birth. Your study doctor will report the pregnancy, the progress, and the outcome to Bristol-Myers Squibb (BMS).

BMS will NOT pay for routine medical care relating to your pregnancy.

**Thank you for reading this question thoroughly, you will be able to move on to the next page after two minutes.**

[SHOW IF P_COND=2]

## DISPLAY_BENEFITS_T

# **Benefits to You**

**6.1 Are there any benefits to being in this study?**

Being a part of this study may or may not help you. The study drug, Astraxo, could prevent you from having another stroke.

It is also possible that being in this study will **not** give you any benefits, and your health might not change. In some cases, your health could even get worse.

This study may help patients prevent strokes in the future.

[SHOW IF P_COND=2]

DISPLAY_COSTS_T.

# **Costs and Getting Paid Back**

**7.1 Will I be paid for being in this study?**

**No**, you will not be paid to be in this study.

You will not get rights to — or money for — any discovery, invention, or patent that may come out of this study. This form is not a contract between you and Bristol-Myers Squibb (BMS).

**7.2 Will it cost me anything to be in this study?**

There is no direct cost to you to be in this study, but there may be some costs related to the study that you will need to pay. For example:

- Travel costs for visits with your study doctor
- You and your insurance company may have to pay for any standard treatment test or procedure that is not required for this study

You will **not** need to pay for:

- Astraxo, Asprin, or Clopidogrel while you are in the study
- Study visits
- Medical tests needed for this study that you would not get otherwise

**7.3 Will I be paid back for costs related to the study?**

No. You will not be paid back for travel to see your study doctor.

[SHOW IF P_COND=2]

## DISPLAY_LEAVINGSTUDY_T

# **Leaving the Study**

**8.1 Can I leave the study?**

Yes. Taking part in the study is your choice. Even if you decide to join the study now, you can leave at any time. You can also stop getting Astraxo and Aspirin and Clopidogrel but continue with study visits to provide information to your study team.

Contact your study doctor if you want to leave the study.

Bristol-Myers Squibb (BMS) will still use information and samples that have already been collected from you.

**Options for leaving the study:**

| **How you leave the study** | **Study visits, collecting information and samples** | **Collection of information about you from your study doctor or other sources** |
| --- | --- | --- |
| **Option 1:**Stop taking the study drugs but continue follow-up visits | Yes | Even if you withdraw consent for further follow-up or contacts, your study doctor or another person or company hired by Bristol-Myers Squibb (BMS) may continue to collect information on your health status where the law allows, including asking your personal doctor. This information will be used to learn whether Astraxo is safe and works to prevent strokes and to tell government agencies about what happened to people in the study. |
| **Option 2:** Stop taking study drugs and stop all study visits | No |  |

**8.2 Can someone else stop me from being in the study?**

Your study doctor, Bristol-Myers Squibb (BMS), the Food and Drug Administration (FDA) or the Institutional Review Board (IRB) may take you off Astraxo or have you leave the study at any time. This could be because:

- Your study doctor decides that it is best for your health to leave the study
- You are having side effects that are hard to treat
- The study stops early
- You or your partner (for males as assigned at birth) gets pregnant
- You cannot make your visits or follow study rules
- Bristol-Myers Squibb (BMS) stops working on Astraxo

If you leave the study completely because of your own free choice or because someone else has taken you off, you will no longer get Astraxo.

**Thank you for reading this question thoroughly, you will be able to move on to the next page after one minute.**

[SHOW IF P_COND=2]

## DISPLAY_AFTERSTUDY_T

# **After the Study**

**9.1 Can I get the study drug after the study is over?**

**No, you cannot.** After you finish the study, your study doctor will ask you to return all left over pills you have. Your study doctor will talk with you about other options to treat your condition before you finish the study.

[SHOW IF P_COND=2]

## DISPLAY_RESPONSIBILITIES_T

# **Your Responsibilities**

**10.1 What do you need to do to be in this study?**

Before you start the study:

- Tell the study doctor about all the treatments you are currently using — including prescription medicines, over-the-counter drugs, vitamins, herbal supplements, and creams/ointments
- Ask your study doctor if there are any drugs that you should not take during the study

During the study:

- Go to all your study visits and follow all study rules
- Carefully follow the study team’s instructions for taking the study drugs, Astraxo, Aspirin and Clopidogrel
- Do not join any other study unless your study doctor says it is okay

Tell the study doctor and study team if:

- You cannot make a visit
- You are having any side effects
- You start any new drugs or treatments that are not part of the study
- You get pregnant or get your partner pregnant
- Your address, telephone number, or other contact information changes

Also, be sure to tell your personal doctors and other important members of your health care team that you are joining the study.

Bristol-Myers Squibb (BMS) will give you a Subject Alert Card (SAC). This card will explain this study to any doctor who is not part of the study who may provide you care. **Keep this card with you at all times.**

**Thank you for reading this question thoroughly, you will be able to move on to the next page after one minute.**

[SHOW IF P_COND=2]

## DISPLAY_MISSVISIT_T

**10.2 What happens if I miss my study visits?**

If you miss your visits, your study team will try to contact you. If your study team cannot reach you, they may ask another person or hire a company to help find you or find information about your health. To do that, your study team will give the company some information about you, like your name and last address. Then the company may search the internet and other places for information about you.

Only your study team will contact you directly.

[SHOW IF P_COND=2]

## DISPLAY_DATACOLLECTION_T

# **Data Collection and Privacy**

**11.1 How will my name and identity be kept private?**

Your privacy is very important. Bristol-Myers Squibb (BMS) and your study team will take steps to protect your personal information as required by law. Only for the purpose of this study, your personal information collected will be shared with others as indicated in this informed consent form.

**11.2 What information will the study team collect from me?**

The study team will collect information and samples. This might include your:

- Name
- Address
- Medical history
- Test results
- Relevant biological samples (like a blood test)

The study will **not** use your samples to map your DNA.

Your study team will replace personal information that could identify you (like your name) with a special code that only the team knows. They will also store any information that could link you or your name to the code in a secure way at the study site. This way, no one who looks at the information will be able to identify you. This information is called coded data.

**11.3 Who can see my health information? How will the study team use it?**

If you agree to be in the study, some people and groups will be able to see information that could identify you for safety purposes and to make sure that the study is running correctly. This includes:

- National and international government agencies in different countries, like the United States Food and Drug Administration (FDA) or the European Union European Medicines Agency (EMA)
- Ethics committees that are responsible for overseeing the study
- Bristol-Myers Squibb (BMS) and those working for or with Bristol-Myers Squibb (BMS) for the purpose of ensuring the quality and safety of the study.

Bristol-Myers Squibb (BMS) may use your coded data to:

- Understand if the study drug works and is safe
- Check if the study is running correctly
- Report side effects and other risks
- Support health information provided to government groups, like the FDA or EMA
- Support internal research and development related to the study drug and disease or answer other questions that may help advance drug development and scientific research involving the study drug and disease or related diseases
- Write articles or presentations (writers will never share your identity) or be included in collections of other research information
- Develop new tests related to the study drug and the disease
- Share with partners, vendors, or labs helping with research
- Share with collaborators for further scientific research

Bristol-Myers Squibb (BMS) usually stores health information for 25 years, or longer if the law requires. BMS may keep health information that does not identify you permanently.

**Thank you for reading this question thoroughly, you will be able to move on to the next page after two minutes.**

[SHOW IF P_COND=2]

## DISPLAY_INJURYILLNESS_T

# **Study-Related Injury or Illness**

**12.1 If I get hurt or sick during the study, will my medical costs be covered?**

If you think you have been hurt or become sick, please get medical care right away and let your study team know.

Bristol-Myers Squibb (BMS) will ensure that your medical costs are paid for if you experience an injury or illness that was caused by the drugs in this study or a badly done test.

You will not lose any of your legal rights by signing and dating this consent form.

[SHOW IF P_COND=2]

## DISPLAY_MOREINFO_T

# **Where to Find More Information**

**13.1 Where can I go to find more information?**

Your own research results will not be given back to you.

General information about the study and a summary of the study results will be available at ClinicalTrials.gov, as required by U.S. law. The description will not include any information that can identify you. You can search this website at any time.

**13.2 Who do I contact if I have questions?**

|  | **Questions about the study** | **Questions about your rights** | **Questions about a study- related injury or illness** |
| --- | --- | --- | --- |
| **Name** | Dr. John Smith | Kevin Williams | Dr. John Smith |
| **Role** | Study Doctor | IRB Representative | Study Doctor |
| **Address** | 1 Convention Dr | 423 Guardian Ave | 1 Convention Dr |
| **Contact** | smith@bms.com | williams@irb.com | smith@bms.com |

[SHOW IF P_COND=2]

## DISPLAY_CONSENTTOJOIN_T

**Consent to Join the Study**

- The study team took the time to answer all my questions.
- I got a Subject Alert Card (SAC) with my study doctor’s contact information.
- I understand that being part of the study may not directly benefit me, but it could help develop treatments for other patients in the future.
- I choose to take part in this study and to take all the tests and drug(s).
- I understand my personal doctor may be told I am joining this study.
- I am allowing the use of my personal information, my medical records, and coded data.
- I will not get any money from new drugs or information that is developed or discovered based on this study.
- I can leave the study at any time, and I will not be penalized or lose any benefits.
- I have read this informed consent form and I understand it.
- I have not entered into a contract, and I am not waiving my legal rights by signing this informed consent form.
- I will get a copy of this informed consent form.

[SHOW IF P_COND=3]

## DISPLAY_GOAL_S

**Project Goal**

Informed consent forms are important documents used in clinical studies. These forms tell the patient considering whether to join a study about general tests and procedures, risks and side effects, and their right to leave the study.

Using a modified informed consent form, this study will measure your comprehension, satisfaction, and willingness to join a possible drug study. We hope these findings will inform researchers on how to improve the informed consent form and entire process.

[SHOW IF P_COND = 3]

## DISPLAY_INSTRUCTIONS_S.

**Instructions**

We are asking you to help improve the informed consent form that patients will read before participating in a drug testing study. Before deciding to join the study, a patient would read this informed consent form.

Imagine you are one of these patients. You will read the informed consent form and answer some questions. Be aware that the form might be long; it could take you about 10 minutes to read. Many of the pages are time-restricted to ensure that you have read everything.

After reading the form, you will answer about 30 short questions. We expect this part to take a maximum of 5 to 10 minutes.

We suggest that you take this survey **only on a laptop or computer** and **when you have 15 minutes,** to avoid interruptions or distractions. If that isn’t now, feel free to close the survey and re-enter it later.

**Thank you for reading this question thoroughly, you will be able to move on to the next page after one minute.**

[SHOW IF P_COND = 3]

## DISPLAY_INFO_F.

**Thank you for reading this question thoroughly, you will be able to move on to the next page after one minute.**

[SHOW IF P_COND = 3]

DISPLAY_RISK_F.

**Thank you for reading this question thoroughly, you will be able to move on to the next page after two minutes.**

[SHOW IF P_COND = 3]

## DISPLAY_TREAT_F.

**Thank you for reading this question thoroughly, you will be able to move on to the next page after two minutes.**

DISPLAY_TRANS.

Thank you for carefully reading the informed consent form. We now have some questions for you. These will take no more than 10 minutes of your time.

Q1.

Overall, how well informed do you feel about the study?

RESPONSE OPTIONS:

1. Very well informed
2. Well informed
3. Not well informed
4. Very poorly informed

Q2.

Have you received all of the information about the study that you want?

RESPONSE OPTIONS:

1. Yes
2. No
77. Not sure

[SHOW IF Q2 = 2 OR 77]

Q3.

What topics would you like more information about?

*Please select all that apply*

RESPONSE OPTIONS:

1. Risks
2. Benefits
3. Purpose of the study
4. Follow up visits
5. Things that will be done during the study
6. Something else, please specify: [TEXTBOX]

Q4.

Which of the following best describes Astraxo?

RESPONSE OPTIONS:

1. A not approved study drug that, with other drugs, might work to prevent future strokes for patients who have had a stroke before
2. A not approved study drug that, by itself, might prevent future strokes for people who have had a stroke before
3. An approved drug that kills blood clotting cells to prevent strokes
4. An approved stroke preventing drug used at a higher dose for people at high risk of having a stroke
77. Not sure

Q5.

What is the main purpose of this study? To find out:

RESPONSE OPTIONS:

1. How the drug Astraxo alone prevents strokes for patients
2. The highest dose of clopidogrel and Astraxo that patients who have had a stroke can tolerate
3. If clopidogrel and Astraxo increase the risk of stroke
4. How the drug Astraxo combined with other drugs can prevent future strokes
77. Not sure

Q6.

When are patients in the study allowed to leave the study?

RESPONSE OPTIONS:

1. Only when the study team says they can leave
2. After they have taken two doses of Astraxo
3. Whenever they want to leave the study
4. When the study ends
77. Not sure

Q7.

Will the study doctor be able to choose the treatment each patient gets in this study?

RESPONSE OPTIONS:

1. Yes, the study team will pick the patients at highest risk of stoke to take Astraxo
2. Yes, the study team will pick the patients who have the best chance of avoiding a stroke to take Astraxo
3. No, treatment is based on only random chance, like flipping a coin or picking a winning lottery number
4. No, because all patients in the study are getting the same treatment
77. Not sure

Q8.

Which of the following organizations may have access to the records of patients like you in the study?

RESPONSE OPTIONS:

1. World Health Organization (WHO)
2. Food and Drug Administration (FDA)
3. Centers for Disease Control (CDC)
4. National Institute of Health (NIH)
77. Not sure

Q9.

Which one of these is a most frequent/very common risk of Astraxo:

RESPONSE OPTIONS:

1. Blurry vision
2. Tingling in the hands and feet
3. Bleeding from the stomach
4. Loose stools
77. Not sure

Q10.

What is a placebo?

RESPONSE OPTIONS:

1. Placebo is getting half a dosage of the study drug in a trial
2. Placebo is a common type of over-the-counter drug combined with Astraxo to prevent strokes
3. Placebo is a not approved drug which you can’t normally buy
4. Placebo is a dummy or sugar pill that has no medicine to prevent strokes in it
77. Not sure

Q11.

Which is a guaranteed benefit for the patients in this study?

RESPONSE OPTIONS:

1. The patients will get improved quality of life
2. The patients will not have a stroke again
3. The patients’ health may or may not benefit, but findings will help future patients
4. The patients’ blood pressure will go down
77. Not sure

Q12.

Where will patients do the tests for the screening part of the study?

RESPONSE OPTIONS:

1. Patients will be mailed the tests by the study team, and they will complete them at home
2. The study team will come to patient homes to do the tests
3. Patients will go to a doctor’s office to do the tests for 2 days in a row
4. Patients will stay overnight in a hospital to do the tests for 2 days in a row
77. Not sure

Q13.

About how long is the whole study in number of days?

RESPONSE OPTIONS:

1. 2 days
2. 30 days
3. 100 days
4. 200 days
5. 365 days
77. Not sure

Q14.

Which of the following organizations is paying for this study?

RESPONSE OPTIONS:

1. National Institute of Health (NIH)
2. World Health Organization (WHO)
3. Pfizer
4. Bristol Myers Squibb (BMS)
77. Not sure

Q15.

Which is NOT a reason a patient might be taken out of the study after they join?

RESPONSE OPTIONS:

1. The patient is having serious side effects from the study drug
2. The patient cannot make study visits or follow study rules
3. The study team finds someone else who needs the study drug more that the patient
4. The study ends early
5. The patient gets someone pregnant or someone gets the patient pregnant
77. Not sure

Q16.

How much are patients going to be paid if they join this clinical study? (Not for taking this survey with AmeriSpeak)

RESPONSE OPTIONS:

1. $50
2. $100
3. $200
4. Patients are not getting paid to be in this clinical study
77. Not sure

Q17.

How long will patients’ health information be stored after the study ends?

RESPONSE OPTIONS:

1. 1 year
2. 10 years
3. 25 years
4. Indefinitely
77. Not sure

**Section 3. Motivation and Previous Treatments**

DISPLAY_MOTIVATION.

For the next section, please answer as yourself, not as a potential patient in the study you read about.

Q18.

If you were told that this study would **not** help you directly but would help other patients, how likely would you be to participate?

RESPONSE OPTIONS:

1. Very likely
2. Likely
3. Neutral
4. Unlikely
5. Very unlikely

Q19.

In the past 5 years, how many medical research studies have you participated in?

RESPONSE OPTIONS:

1. 0
2. 1
3. 2
4. 3 or more
77. Not sure

Q20.

Thinking about the consent form, would you say the length of the consent form was:

RESPONSE OPTIONS:

1. Too long
2. About right
3. Too short

Q21.

Thinking about the consent form, would you say the amount of detail in the form was:

RESPONSE OPTIONS:

1. Too detailed
2. About right
3. Not detailed enough

Q22.

How easy or hard was it for you to understand the information in the consent form for the study?

RESPONSE OPTIONS:

1. Very hard
2. Moderately hard
3. Neutral
4. Moderately easy
5. Very easy

Q23.

On a scale from 0-10, how likely would you be to recommend this study to a patient at risk of stroke?

Q24.

How can we make your experience with this consent process better?

*Please select all that apply.*

RESPONSE OPTIONS:

1. Have a shorter consent form with less details
2. Use video instead of written form
3. Use more pictures, tables and timelines
4. Have less information on preventing pregnancy
5. Have more information on potential benefits
6. Have a longer consent form with more details
7. Have less information on keeping my information private
8. Other: please specify [TEXTBOX]

Q25.

Is there anything else you would like share about how we can make the informed consent process better?

[MEDIUM TEXTBOX]

[DROPDOWN]

Q26.

Please rank the following topics from the form by importance to you where 1 is most important and 10 is least important:

RESPONSE OPTIONS:

A. Side Effects and Other Risks
B. Benefits To You
C. Data Collection and Privacy
D. Treatment
E. Costs and Getting Paid Back
F. Your Responsibilities
G. Study Timeline, Visits, and Tests
H. Where to Find More Information
I. Leaving the Study
J. Study-Related Injury or Illness

Q27.

How do you usually pay for your health care?

RESPONSE OPTIONS:

1. Private health insurance such as Blue Cross Blue Shield
2. Medicare
3. Medicaid
4. Veteran’s Administration (VA)
5. Public health or free clinic
6. Out of pocket with my own money
7. Other, please specify [TEXTBOX]
77. Not sure

Q28.

How carefully did you read the consent form?

RESPONSE OPTIONS:

1. Very carefully
2. Carefully
3. Somewhat carefully
4. Not very carefully
5. I did not read it at all

**Demographic Profile:**

**Additional questions asked of panelists prior to this survey**

**and are included in the survey data**

| Variable | Values |
| --- | --- |
| Gender | 1 = Male |
|  | 2 = Female |
| Age | Age in years |
| Age (7 categories) | 1 = 18-24; 2 = 25-34; 3 = 35-44; 4 = 45-54; 5 = 55-64; 6 = 65-74; 7 = 75+ |
| Age (4 categories) | 1 = 18-29; 2 = 30-44; 3 = 45-59; 4 = 60+ |
| Education (5 categories) | 1 = Less than HS |
|  | 2 = HS graduate |
|  | 3 = Some college/associates degree |
|  | 4 = Bachelor’s degree |
|  | 5 = Post grad study/professional degree |
| Race/Ethnicity | 1 = White, Non-Hispanic |
|  | 2 = Black, Non-Hispanic |
|  | 3 = Other, Non-Hispanic |
|  | 4 = Hispanic |
|  | 5 = 2+ races, Non-Hispanic |
|  | 6 = Asian/Pacific Islander, Non-Hispanic |
| Housing Type | 1 = A one-family house detached from any other house |
|  | 2 = A one-family house attached to one or more houses |
|  | 3 = A building with 2 or more apartments |
|  | 4 = A mobile home or trailer |
|  | 5 = Boat, RV, van, etc. |
| Household Income (18 categories) | 1 = Less than $5,000 2 = $5,000 to $9,999 |
|  | 3 = $10,000 to $14,999 4 = $15,000 to $19,999 |
|  | 5 = $20,000 to $24,999 6 = $25,000 to $29,999 |
|  | 7 = $30,000 to $34,999 8 = $35,000 to $39,999 |
|  | 9 = $40,000 to $49,999 10 = $50,000 to $59,999 |
|  | 11 = $60,000 to $74,999 12 = $75,000 to $84,999 |
|  | 13 = $85,000 to $99,999 14 = $100,000 to $124,999 |
|  | 15 = $125,000 to $149,999 16 = $150,000 to $174,999 |
|  | 17 = $175,000 to $199,999 18 = $200,000 or more |
| Household Income (9 categories) | 1 = Less than $10,000 |
|  | 2 = $10,000 to $19,999 |
|  | 3 = $20,000 to $29,999 |
|  | 4 = $30,000 to $39,999 |
|  | 5 = $40,000 to $49,999 |
|  | 6 = $50,000 to $74,999 |
|  | 7 = $75,000 to $99,999 |
|  | 8 = $100,000 to $149,999 |
|  | 9 = $150,000 or more |
| Household Income (4 categories) | 1 = Less than $30,000 |
|  | 2 = $30,000 to $59,999 |
|  | 3 = $60,000 to $99,999 |
|  | 4 = $100,000 or more |
| Marital Status | 1 = Married |
|  | 2 = Widowed |
|  | 3 = Divorced |
|  | 4 = Separated |
|  | 5 = Never married |
| Metropolitan Statistical Area  Status | 0 = Non-Metro |
|  | 1 = Metro (as defined US OMB Core-Based Statistical Area) |
| Home Internet Access | 0 = No |
|  | 1 = Yes |
| Telephone Service | 1 = Landline telephone only |
|  | 2 = Have a landline, but mostly use cellphone |
|  | 3 = Have cellphone, but mostly use landline |
|  | 4 = Cellphone only |
|  | 5 = No telephone service |
| Ownership of Living Quarters | 1 = Owned or being bought by you or someone in your household |
|  | 2 = Rented for cash |
|  | 3 = Occupied without payment of cash rent |
| Region 4 (US Census) | 1 = Northeast |
|  | 2 = Midwest |
|  | 3 = South |
|  | 4 = West |
| Region 9 (US Census) | 1 = New England |
|  | 2 = Mid-Atlantic |
|  | 3 = East-North Central |
|  | 4 = West-North Central |
|  | 5 = South Atlantic |
|  | 6 = East-South Central |
|  | 7 = West-South Central |
|  | 8 = Mountain |
|  | 9 = Pacific |
| State | State of residence |
| Household Size | Total number of members in household |
| HH members, age 0-1 | Known number of household members in age group |
| HH members, age 2-5 | Known number of household members in age group |
| HH members, age 6-12 | Known number of household members in age group |
| HH members, age 13-17 | Known number of household members in age group |
| HH members, age 18+ | Known number of household members in age group |
| Current Employment Status | 1 = Working - as a paid employee |
|  | 2 = Working - self-employed |
|  | 3 = Not working - on temporary layoff from a job |
|  | 4 = Not working - looking for work |
|  | 5 = Not working – retired |
|  | 6 = Not working – disabled |
|  | 7 = Not working – other |
