# Supplementary material for: A randomized control trial of re-designed and shorter research informed consent forms to improve comprehension
Source: Clin Trials. 2026 Mar 28;23(4):421–9. doi: 10.1177/17407745261429936 (PMC13451594; doi:10.1177/17407745261429936)
Supplement: Supplementary file 2 [file sj-docx-2-ctj-10.1177_17407745261429936.docx]

**Supplement: Table 4 – Percentage of Participants who Answered Correctly Comprehension Question by ICFs**

| **Comprehension Question** | **Overall**  **N (% correct)** | **Long ICF**  **N (% correct)** | **Medium ICF N (% correct)** | **Short ICF**  **N (% correct)** | **p-value** |
| --- | --- | --- | --- | --- | --- |
| 1. Which of the following best describes [the drug]? | 231 (69.2%) | 63 (61.2%) | 82 (76.6%) | 86 (69.4%) | Overall: 0.053 |
| 2. What is the main purpose of this study? | 234 (70.1%) | 66 (64.1%) | 81 (75.7%) | 87 (70.2%) | Overall: 0.180 |
| 3. When are patients allowed to leave this study? | 302 (90.4%) | 91 (88.3%) | 101 (94.4%) | 110 (88.7%) | Overall: 0.240 |
| 4. Will the study doctor be able to choose the treatment each patient gets in this study? | 226 (67.7%) | 59 (57.3%) | 79 (73.8%) | 88 (71.0%) | Overall: 0.023  Long v Medium: 0.017  Long v Short: 0.044  Medium v Short: 0.074 |
| 5. Which of the following organizations may have access to the records of patients like you in the study? | 177 (53.0%) | 47 (45.6%) | 64 (59.8%) | 66 (53.2%) | Overall: 0.120 |
| 6. Which one of these is a most frequent/very common risk of [the drug]: | 176 (52.7%) | 32 (31.1%) | 73 (68.2%) | 71 (57.3%) | Overall: <0.001  Long v Medium: <0.001  Long v Short: <0.001  Medium v Short: 0.110 |
| 7. What is a placebo? | 300 (89.8%) | 87 (84.5%) | 101 (94.4%) | 112 (90.3%) | Overall: 0.058 |
| 8. Which is a guaranteed benefit for the patients in this study? | 297 (88.9%) | 88 (85.4%) | 99 (92.5%) | 110 (88.7%) | Overall: 0.261 |
| 9. Where will patients do the tests for the screening part of the study? | 170 (50.9%) | 65 (63.1%) | 69 (64.5%) | 36 (29.0%) | Overall: <0.001  Long v Medium: 0.950  Long v Short: <0.001  Medium v Short: <0.001 |
| 10. About how long is the whole study in number of days? | 253 (75.7%) | 74 (71.8%) | 95 (88.8%) | 84 (67.7%) | Overall: <0.001  Long v Medium: 0.003  Long v Short: 0.600  Medium v Short: 0.025 |
| 11. Which of the following organizations is paying for this study? | 262 (78.4%) | 64 (62.1%) | 97 (90.7%) | 101 (81.5%) | Overall: <0.001  Long v Medium: <0.001  Long v Short: 0.002  Medium v Short: 0.071 |
| 12. Which is NOT a reason a patient might be taken out of the study after they join? | 217 (65.0%) | 67 (65.0%) | 79 (73.8%) | 71 (57.3%) | Overall: 0.031  Long v Medium: 0.220  Long v Short: 0.290  Medium v Short: 0.013 |
| 13. How much are patients going to be paid if they join this clinical study? | 289 (86.5%) | 80 (77.7%) | 100 (93.5%) | 109 (87.9%) | Overall: 0.003  Long v Medium: 0.002  Long v Short: 0.060  Medium v Short: 0.230 |
| 14. How long will patients’ health information be stored after the study ends? | 196 (58.7%) | 67 (65.0%) | 63 (58.9%) | 66 (53.2%) | Overall: 0.197 |

**Supplement: Table 5 – Univariate and Multivariate Analyses of Scoring Higher on Comprehension**

| **Characteristic** | **No. With Characteristic**  **(%)** | **Overall Comprehension Score**  **(% of 14)** | **Univariate OR of Support**  **(95% CI)** | **Multivariate OR of Support**  **(95% CI)** |
| --- | --- | --- | --- | --- |
| Long ICF | 103 (30.8) | 9.2 (65.7) | 0.62 (0.39 - 1.00) | 0.81 (0.72 - 0.89) |
| Medium ICF | 107 (32.0) | 11.1 (79.3) | 2.73 (1.68 - 4.42) | 1.57 (1.28 - 1.95) |
| Short ICF | 124 (37.1) | 9.7 (69.3) | 0.62 (0.40 - 0.97) | 0.76 (0.7 - 0.89) |
| White, non-Hispanic | 194 (58.1) | 10.6 (75.7) | 1.96 (1.26 - 3.04) | 1.43 (1.23 - 1.83) |
| Black, non-Hispanic | 46 (13.8) | 8.8 (62.9) | 0.55 (0.29 - 1.04) | 0.87 (0.74 - 0.92) |
| Asian, non-Hispanic | 29 (8.7) | 10.3 (73.6) | 1.35 (0.63 - 2.93) | 1.13 (1.04 - 1.4) |
| Hispanic, Latino | 59 (17.7) | 8.5 (60.7) | 0.45 (0.25 - 0.80) | 0.81 (0.47 - 0.85) |
| Other, non-Hispanic | 6 (1.8) | 9.5 (67.9) | 0.41 (0.19 - 0.90) | 0.83 (0.56 - 0.94) |
| Less than high school and high school or equivalent | 125 (37.4) | 8.7 (62.1) | 0.42 (0.27 - 0.66) | 0.64 (0.49 - 0.74) |
| Some college, associates, or bachelor's degree | 152 (45.6) | 10.6 (75.7) | 1.57 (1.02 - 2.42) | 1.16 (1.1 - 1.26) |
| Post graduate degree or professional degree | 57 (17.1) | 11.0 (78.6) | 1.92 (1.06 - 3.47) | 1.31 (1.2 - 1.73) |
| Insurance - Private | 168 (50.3) | 10.3 (73.6) | 1.05 (0.68 - 1.61) | 1.01 (0.93 - 1.2) |
| Insurance - Government Program | 135 (40.4) | 10.1 (72.1) | 1.29 (0.83 - 2.00) | 1.21 (1.09 - 1.65) |
| Insurance - Other | 31 (9.3) | 7.7 (55.0) | 0.41 (0.19 - 0.90) | 0.83 (0.56 - 0.94) |

**Supplement: Table 6 – Areas of Improvement by ICFs**

| **Response** | **Overall**  **N (%)** | **Long ICF**  **N (%)** | **Medium ICF**  **N (%)** | **Short ICF**  **N (%)** | **p-value** |
| --- | --- | --- | --- | --- | --- |
| More Information on:  Things that will be done during the study | 31 (9.3) | 3 (2.9) | 10 (9.3) | 18 (14.5) | Overall: 0.01  Long v Medium: 0.10  Long v Short: 0.01  Medium v Short: 0.01 |
| Risks | 29 (8.7) | 5 (4.9) | 12 (11.2) | 12 (9.7) | Overall: 0.23 |
| Benefits | 24 (7.2) | 3 (2.9) | 12 (11.2) | 9 (7.3) | Overall: 0.07 |
| Follow up visits | 17 (5.1) | 3 (2.9) | 5 (4.7) | 9 (7.3) | Overall: 0.32 |
| Something else | 15 (4.5) | 2 (1.9) | 7 (6.5) | 6 (4.8) | Overall: 0.27 |
| Purpose of the study | 14 (4.2) | 2 (1.9) | 6 (5.6) | 6 (4.8) | Overall: 0.37 |
| Use more pictures, tables and timelines | 142(42.5) | 52 (50.5) | 41(38.3) | 49 (39.5) | Overall: 0.14 |
| Have more information on potential benefits | 128 (38.3) | 39 (37.9) | 42 (39.3) | 47 (37.9) | Overall: 0.97 |
| Use video instead of written form | 118 (35.3) | 43 (41.7) | 37 (34.6) | 38 (30.6) | Overall: 0.22 |
| Have a shorter consent form with less details | 50 (15) | 24 (23.3) | 12 (11.2) | 14 (11.3) | Overall: 0.02  Long v Medium: 0.03  Long v Short: 0.03  Medium v Short: 1.00 |
| Have less information on preventing pregnancy | 39 (11.7) | 21 (20.4) | 13 (12.1) | 5 (4.0) | Overall: <0.001  Long v Medium: 0.15  Long v Short: <0.001  Medium v Short: 0.04 |
| Other | 27 (8.1) | 4 (3.9) | 14 (13.1) | 9 (7.3) | Overall: 0.05  Long v Medium: 0.03  Long v Short: 0.42  Medium v Short: 0.21 |
| Have less information on keeping my information private | 21(6.3) | 14 (13.6) | 5 (4.7) | 2 (1.6) | Overall: <0.001  Long v Medium: 0.04  Long v Short: <0.001  Medium v Short: 0.33 |
| Have a longer consent form with more details | 14 (4.2) | 3 (2.9) | 2 (1.9) | 9 (7.3) | Overall: 0.09 |

**Supplement: Table 7 – Overall Comprehension Score Across ICFs, Stratified by Sociodemographic Characteristic**

| **Characteristic** | **Classification** | **Overall**  **Mean Score n out of 14**  **(% of 14)** | **Long ICF**  **Mean Score**  **n out of 14**  **(% of 14)** | **Medium ICF**  **Mean Score**  **n out of 14**  **(% of 14)** | **Short ICF Mean Score**  **n out of 14**  **(% of 14)** | **p-value** |
| --- | --- | --- | --- | --- | --- | --- |
| **Age** | 18-44 | 9.4 (67.1) | 8.1 (57.9) | 11.1 (79.3) | 9.1 (65.0) | Overall: 0.002  Long v Medium: 0.002  Long v Short: 0.420  Medium v Short: 0.060 |
|  | 45-64 | 10.2 (72.9) | 9.5 (67.9) | 10.8 (77.1) | 10.1 (72.1) | Overall: 0.280 |
|  | 65+ | 10.5 (75.0) | 10.6 (75.7) | 11.4 (81.4) | 9.5 (67.9) | Overall: 0.053 |
| **Sex** | Male | 10.0 (71.4) | 9.2 (65.7) | 11.2 (80.0) | 9.7 (69.3) | Overall: 0.009  Long v Medium: 0.010  Long v Short: 0.720  Medium v Short: 0.060 |
|  | Female | 9.9 (70.7) | 9.3 (66.4) | 10.9 (77.9) | 9.6 (68.6) | Overall: 0.034  Long v Medium: 0.040  Long v Short: 0.850  Medium v Short: 0.110 |
| **Race / Ethnicity** | White, non-Hispanic | 10.6 (75.7) | 9.8 (70.0) | 11.8 (84.3) | 10.3 (73.6) | Overall: 0.003  Long v Medium: 0.004  Long v Short: 0.710  Medium v Short: 0.020 |
|  | Black, non-Hispanic | 8.8 (62.9) | 9.1 (65.0) | 9.6 (68.6) | 8.3 (59.3) | Overall: 0.620 |
|  | Asian, non-Hispanic | 10.3 (73.6) | 9.4 (67.1) | 12.5 (89.2) | 9.6 (68.6) | Overall: 0.053 |
|  | Hispanic/Latino | 8.5 (60.7) | 7.3 (52.1) | 9.5 (67.9) | 8.8 (62.8) | Overall: 0.140 |
|  | Other, non-Hispanic | 9.5 (67.9) | 11.3 (80.7) | 7.7 (55.0) | NA | NA |
| **Education** | High school equivalent or less | 8.7 (62.1) | 8.0 (57.1) | 9.4 (67.1) | 8.8 (62.8) | Overall: 0.290 |
|  | Some college, associates, or bachelor’s degrees | 10.6 (75.7) | 10.2 (72.9) | 11.7 (83.6) | 10.0 (71.4) | Overall: 0.004  Long v Medium: 0.032  Long v Short: 0.890  Medium v Short: 0.004 |
|  | Post graduate degree or professional degree | 11.0 (78.6) | 9.9 (70.7) | 12.4 (88.6) | 10.6 (75.7) | Overall: 0.032  Long v Medium: 0.031  Long v Short: 0.710  Medium v Short: 0.140 |
| **Household Income** | Less than $25,000 | 9.1 (65.0) | 9.5 (67.8) | 8.3 (59.2) | 9.2 (65.7) | Overall: 0.810 |
|  | $25,000-$49,999 | 9.4 (67.1) | 8.7 (62.1) | 10.5 (75.0) | 9.1 (65.0) | Overall: 0.210 |
|  | $50,00-$99,999 | 10.2 (72.9) | 9.3 (66.4) | 11.3 (80.7) | 9.9 (70.7) | Overall: 0.027  Long v Medium: 0.029  Long v Short: 0.700  Medium v Short: 0.110 |
|  | $100,000 or more | 10.4 (74.3) | 9.4 (67.1) | 11.6 (82.9) | 10.0 (71.4) | Overall: 0.010  Long v Medium: 0.013  Long v Short: 0.710  Medium v Short: 0.058 |
| **Geography** | Urban | 10.1 (72.1) | 9.3 (66.4) | 11.7 (83.6) | 9.7 (69.3) | Overall: 0.003  Long v Medium: 0.003  Long v Short: 0.820  Medium v Short: 0.021 |
|  | Suburban | 10.1 (72.1) | 9.6 (68.6) | 11.0 (78.6) | 9.7 (69.3) | Overall: 0.069 |
|  | Rural | 9.2 (65.7) | 8.4 (60.0) | 9.9 (70.7) | 9.5 (67.9) | Overall: 0.520 |
| **Region** | Northeast | 10.6 (75.7) | 10.5 (75.0) | 12.0 (85.7) | 10.0 (71.4) | Overall: 0.170 |
|  | South | 9.3 (66.4) | 8.1 (57.9) | 11.1 (79.3) | 8.7 (62.1) | Overall: 0.002  Long v Medium: 0.003  Long v Short: 0.790  Medium v Short: 0.015 |
|  | Midwest | 10.4 (74.3) | 9.2 (65.7) | 11.4 (81.4) | 10.4 (74.3) | Overall: 0.110 |
|  | West | 10.0 (71.4) | 9.6 (68.6) | 10.5 (75) | 9.9 (70.7) | Overall: 0.580 |
| **Form of Health Insurance** | Private | 10.3 (73.6) | 9.5 (67.9) | 11.3 (80.7) | 10.0 (71.4) | Overall: 0.011  Long v Medium: 0.010  Long v Short: 0.600  Medium v Short: 0.083 |
|  | Government Program | 10.1 (72.1) | 10.2 (72.9) | 11.0 (78.6) | 9.2 (65.7) | Overall: 0.037  Long v Medium: 0.480  Long v Short: 0.390  Medium v Short: 0.028 |
|  | Other | 7.7 (55.0) | 5.6 (40.0) | 9.6 (68.6) | 9.2 (65.7) | Overall: 0.075 |
